# Analysis of global prevalence of antibiotic resistance in *Acinetobacter baumannii* infections disclosed a faster increase in OECD countries

**DOI:** 10.1038/s41426-018-0038-9

**Published:** 2018-03-14

**Authors:** Ruiqiang Xie, Xiaohua Douglas Zhang, Qi Zhao, Bo Peng, Jun Zheng

**Affiliations:** 1 Faculty of Health Sciences, University of Macau, Macau SAR, China; 2 State Key Laboratory of Bio-Control, School of Life Sciences, Sun Yat-sen University, 510006 Guangzhou, China; 3 Laboratory for Marine Biology and Biotechnology, Qingdao National Laboratory for Marine Science and Technology, 266071 Qingdao, China

## Abstract

*Acinetobacter baumannii* is one of the most challenging nosocomial pathogens due to the emergence and widespread of antibiotic resistance. We aimed to provide the first analysis of global prevalence of antibiotic resistance in *A. baumannii* infections, by synthesizing data and knowledge through a systematic review. We searched studies reporting antibiotic resistance in *A. baumannii* infections using the Medline, Embase, Web of Science, and Cochrane databases from January 2000 to December 2016. Studies were eligible if they investigated and reported antibiotic resistance in *A. baumannii* infections with inpatients or outpatients in hospital. Our investigation showed a high prevalence of resistance to the common prescribed antibiotics in *A. baumannii* infections in both OECD (Organization for Economic Co-operation and Development) and non-OECD countries. Strikingly, though OECD countries have substantially lower pooled prevalence of resistance compared to non-OECD countries based on the data during 2006–2016, a further investigation in a time scale disclosed a faster increase in OECD countries during the past 11 years, and currently both of them have a comparable prevalence of resistance (2011–2016). Tigecycline and colistin are still active but their resistances are expected to become common if the preventative measures are not taken. Antibiotic resistance in *A. baumannii* infection developed fast and is a crisis for both OECD and non-OECD countries. A “post-antibiotic era” for *A. baumannii* infection is expected in the next 10–20 years without immediate actions from pharmaceutical companies and governments.

## Introduction

Antimicrobial resistance (AMR) has become a global crisis due to escalating evolution of resistance coupled with a diminished antibiotic pipeline. A recent high profile report estimates that, by 2050, 10 million people will die from AMR per year if the current situation continues uncontrolled^1^. However, this prediction was challenged due to the lack of detailed data on AMR burden, its morbidity and mortality, the modeling of future scenarios, etc^2^. Therefore, accessing the global prevalence of antibiotic resistance of the pathogens and investigating its developmental trend will aid our understanding of the current situation as well as make proper estimation of future scenario. Thus, this will be highly important for policy making.

The microorganisms that are mainly involved in antibiotic resistance are the so called ESKAPE pathogens, standing for *Enterococcus faecium*, *Staphylococcus aureus*, *Klebsiella pneumoniae*, *Acinetobacter baumannii*, *Pseudomonas aeruginosa*, and *Enterobacteriaceae*, capable of “escaping” from common antibacterial treatments^3^. *A. baumannii* is one of the most challenging pathogens among them due to its particular antibiotic resistance characteristics. World Health Organization has recently published its first ever list of antibiotic resistant “priority pathogens” to secure and guide research and development related to new antibiotics, among which *A. baumannii* was being selected as priority 1 (critical), highlighting its serious threats to public health^4^. *A. baumannii* is an opportunistic Gram-negative bacterium and responsible for a broad range of nosocomial infections, the most important of which are ventilator-associated pneumonia and bloodstream infections, and the mortality rates can reach 35%^5^. It is particularly problematic due to the frequency of multi-drug resistance (MDR) and the high epidemic potential^6^. Currently, *A. baumannii* has developed resistance to almost all known antibiotics, and the MDR has been widely documented^7^. On the other hand, the emergence and widespread of antibiotic resistance have diminished the options of effective therapeutic drug for *A. baumannii* infection, and a clinician has to choose the previously abandoned antibiotic colistin, which is generally associated with more serious adverse effect^8^. Most importantly, it was reported that clinical isolates resistant to colistin have emerged in certain geographical areas^9^, making the last resort of antibiotics in human medicine ineffective.

Given the epidemic potential of antibiotic resistant *A. baumannii*, it is important to know the current prevalence of antibiotic resistance and its developmental trend, the latter of which is critical for the estimation of future scenario. However, the previous studies on the prevalence of antibiotic resistance were largely limited to either certain country, certain timeframe, selected antibiotics, or lack of detailed information and systematic analysis. Hence, we sought to evaluate the global prevalence of pooled resistance in *A. baumannii* infections to the most commonly prescribed antibiotics in hospital by summarizing data identified in the published literatures based on OECD (Organization for Economic Co-operation and Development) status. Antibiotics are obtained mostly only by prescription in the more developed OECD countries, whereas many antibiotics can be purchased over counters without the need for a prescription in developing non-OECD countries^10,11^.

## Methods and materials

### Search strategy and selection criteria

The Medline, Embase, Web of Science, and Cochrane databases were systematically searched for references published from January 2000 to December 2016 without language restrictions. The Medical Subject Heading terms in the literature retrieval were “drug resistance”, “antimicrobial resistance”, and “bacterial resistance”, and searched with different combinations of other key text words. We also searched the relevant patents, websites, conference proceedings, government and national report, and open access material through Web of Science and Google Scholar. We manually checked the reference lists of the original key studies (studies cited by multiple articles) to identify other potential studies. Our search was restricted to *A. baumannii* studies only and has excluded the data of *A. baumannii* complex. The detailed search strategy is provided in Supplementary Table S1.

Two reviewers (RX, JZ) independently screened all the titles and abstracts. We obtained the full-text papers that fulfill the inclusion criteria for evaluation. The studies were strictly filtered and screened with the following eligible standards: the tested *A. baumannii* isolates in the included studies were collected from infected patients in hospitals. Only bacterial isolates clearly being identified as *A. baumannii* were included. The antimicrobial susceptibility testing was following eligible guidelines. The antibiotic resistance was explored, tested, and confirmed in hospital or related research participating laboratories or institutions.

The commonly prescribed antibiotics included in this study were: carbapenems (imipenem or meropenem), amikacin, ampicillin-sulbactam, tobramycin, ceftazidime, piperacillin-tazobactam, cefepime, colistin, and tigecycline^12^. Fluoroquinolones (ciprofloxacin) were not included in this analysis due to either the nearly saturated antibiotic resistance or the lack of enough data (Supplementary Figure S1 and Supplementary Table S2). The MDR *A. baumannii* was defined as resistance to three or more classes of antimicrobial agents including aminoglycosides, β-lactams (excluding the intrinsic resistant agents: penicillin and cephalosporins), carbapenems, fluoroquinolones, and tetracyclines.

### Data extraction and quality evaluation

Two reviewers independently retrieved and evaluated data from the selected studies. The following information were extracted: journal, author, publication year, article design, article country, economic situation, recruited patients, recruitment location and time, antimicrobial susceptibility testing, species identification, antimicrobial agents, and antibiotic sensitivities. The extracted results were stratified by OECD status of the studies following previous meta-analysis^13^. OECD countries often have strong economic vitality and are usually regarded as “developed” countries, whereas non-member countries are regarded as “developing” countries.

The Cochrane collaboration risk of bias tool was carried out to assess the quality of studies by two independent reviewers. We applied the Critical Appraisal Skills Program checklist (http://www.casp-uk.net/) to assess the selection bias for the cohort and case–control papers. The quality assessment charts were generated for “good,” “adequate,” and “poor” reporting based on the Cochrane recommended traffic light system^14^.

### Data synthesis and analysis

The statistical analyses were carried out with the package Metafor in R software and the whole process for this systematic review and meta-analysis followed the PRISMA guideline^15,16^. This package integrates a collection of functions that allow the user to fit fixed or random effects models to implement meta-regression analysis. The 95% confidence interval (CI) for the estimates of resistant *A. baumannii* was calculated. We used forest plots to show the pooled prevalence of resistance for each antibiotic. The pooled prevalence of antibiotic resistance data, measured based on the OECD status of the studies, were characterized with forest plots for each antibiotic, along with 95% CI. The pooled prevalence of drug resistance for each antibiotic was explored and investigated in all the study countries. Furthermore, the pooled prevalence of antibiotic resistance at different time interval was investigated and compared. The heterogeneity was assessed by the *I*^2^ statistics, where *I*^2^ values suggested the level of heterogeneity. The pooled prevalence of antibiotic resistance for each antibiotic was analyzed by three periods (years of 2000–2005, 2006–2010, and 2011–2016), stratified by OECD status. The random-effect meta-analysis was implemented to analyze these studies, and the pooled odds ratios were calculated for each period. Finally, we constructed a meta-regression model to quantify the variation of resistant estimates for each antibiotic between different periods using the odds ratios.

## Results

### Study characteristics and quality of the included studies

**Fig. 1:**
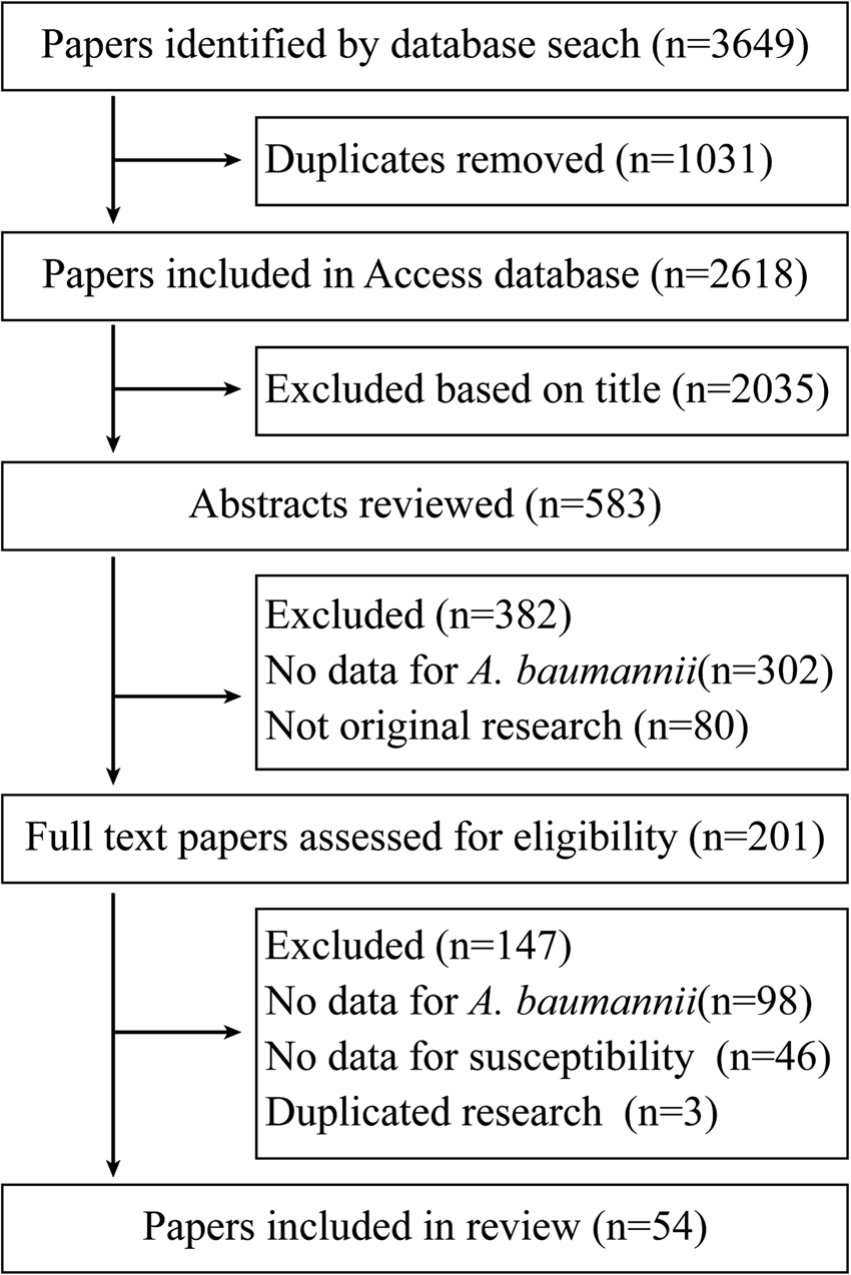
Flow diagram of study selection

**Fig. 2:**
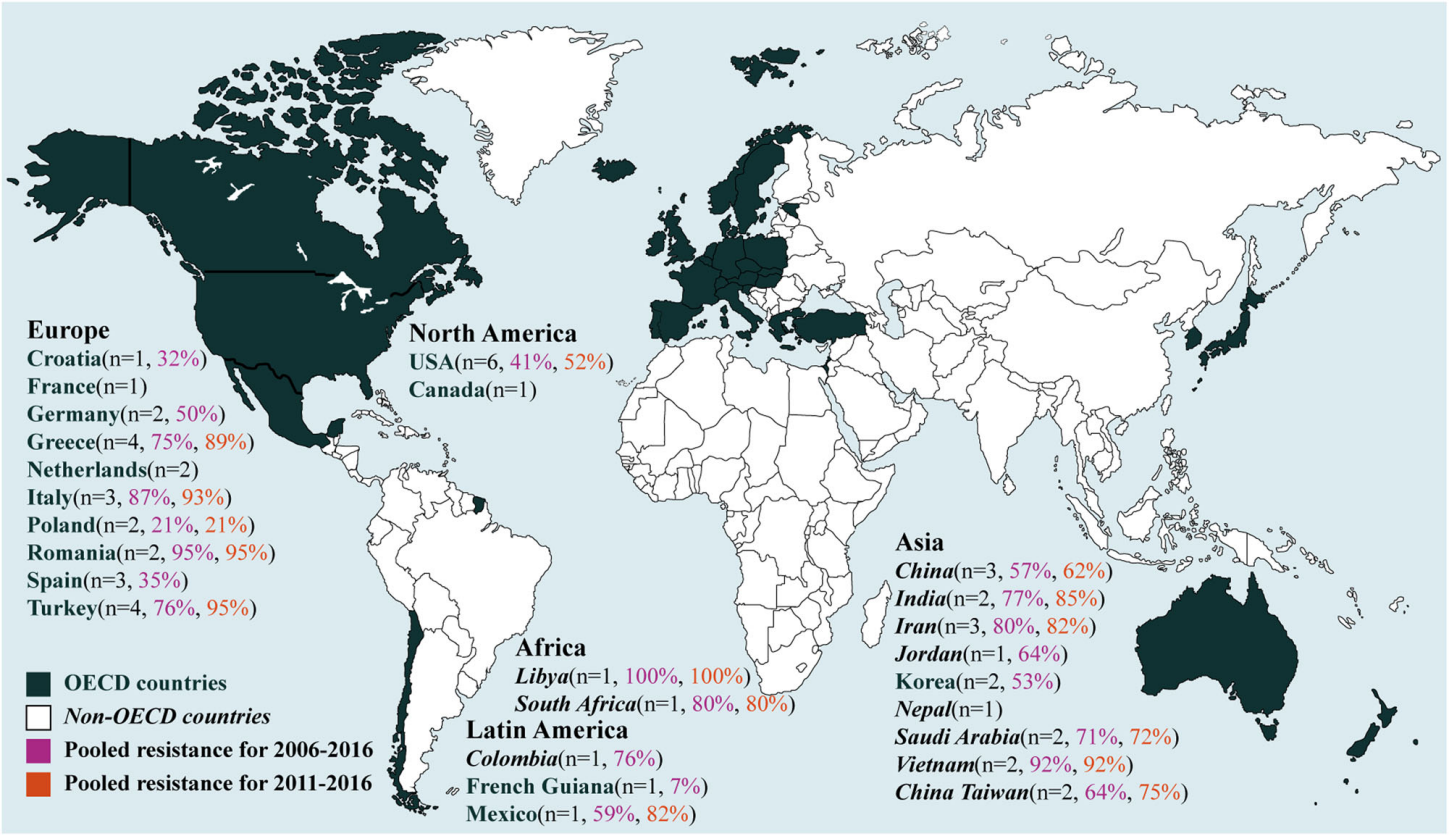
Geographical distribution of the prevalence of antibiotics resistance in *A. baumannii* infections to imipenem (%) by two periods. *n* is the number of included studies per country

We identified 54 studies in our analysis. The detailed reference retrieval process and study assessment were shown in Fig. 1. The characteristics of these studies were summarized in Table 1 and Fig. 2. Thirty five studies from OECD countries reported resistance of 57,188 isolates and 19 studies from non-OECD countries reported 7395 isolates. Antimicrobial sensitivity testing and breakpoints for all antibiotics was detailed in Supplementary Table S2, and for tigecycline and colistin were provided in Supplementary Table S3 and Supplementary Table S4. The breakpoints for tigecycline were mainly according to the European Committee on Antimicrobial Susceptibility Testing (EUCAST) and US Food and Drug Administration (FDA) criteria (Supplementary Table S3). The *A. baumannii* species identification was detailed in Supplementary Table S2.

**Table 1:** The characteristics of included papers on antibiotic resistance of *A. baumannii* infections by OECD status of each country

Study characteristics	No of papers from OECD countries (*n* = 35/supplementary reference)	No of papers from non-OECD countries (*n* = 19/supplementary reference)
Study design		
Retrospective observational	25^1,4,7,8,12,13,15–19,21–32,34,35^	18^36–39,41–54^
Prospective observational	5^2,6,9,14,20^	1^40^
Case–control	3^3,5,10^	0
Cross-sectional	2^11,33^	0
Antibiotic susceptibilities reported		
Imipenem	31^2–9,11–15,18–35^	18^36–48,50–54^
Amikacin	29^3–14,16–25,27,28,30–34^	18^36–45,47–54^
Ampicillin-sulbactam	14^7,8,11,13,15,18,22,24,25,27–29,31,34^	7^36,38,42,44,48,53,54^
Tobramycin	14^5,9,10,12–15,17,19,20,22,25,28,31^	6^39,42,43,47,52,54^
Ceftazidime	27^2–9,11,12,14,15,17–24,27–30,32,34,35^	17^36-49,51,52,54^
Meropenem	26^4,6–9,11,13–20,22–24,26–29,31–35^	16^37–41,43-47,49–54^
Piperacillin-tazobactam	26^2,4–10,12,13,15–21,24,25,27–30,32,34,35^	12^36–38,40–42,44,45,48,52–54^
Cefepime	23^4,6–13,15–19,23–25,27,29,30,32,34,35^	14^36–39,41–46,48,51,52,54^
Colistin	17^3,13,16–20,22,24–27,30–34^	13^39,40,42–50,52,53^
Tigecycline	7^8,16,18,20,24,25,28^	9^40,42,44,46–49,52,53^
MDR	10^7,15–18,21,24,25,28,31^	4^37,42,48,54^
Method of antimicrobial susceptibility testing		
Standard susceptibility testing methods	6^3,4,8,9,13,31^	4^38,39,41,45^
Disk diffusion	8^6,12,14,22,25,27,28,30^	10^25,37,40,42,45,47–49,52,53^
Broth microdilution	10^2,4,7,11,15,16,18,27,32,33^	4^44,47,50,54^
Vitek2	16^1,5,10,15,17,19–21,24–29,33^	5^36,37,46,51,54^
Agar dilution	0	2^36,44^
Etests	6^24,25,28,30,33,34^	4^36,43,51,52^
Sensititre ARIS® 2×	1^23^	0
Guidelines used to interpret antimicrobial sensitivities		
CLSI	30^1–4,6–9,13,14,16–35^	17^36–45,47–51,53,54^
EUCAST	5^20,26,27,33,34^	3^42,47,49^
DIN	1^15^	0
CA-SFM	0	2^12,52^
Not reported	3^5,10,11^	1^46^

*CLSI* The Clinical and Laboratory Standards Institute, *EUCAST* the criteria of the European Committee on Antimicrobial Susceptibility Testing (EUCAST) for Enterobacteriaceae, *DIN* German Institute for Standardization, *CA-SFM* Antibiogram Committee of the French Society for Microbiology

The assessment of the study quality was carried out based on the Cochrane collaboration risk of bias tool. The overall quality of the reported studies was generally good for the key risk of bias (Supplementary Figure S2).

### The pooled prevalence of antibiotic resistance

During our initial analysis of the resistant status, we found limited data in non-OECD countries before 2005, suggesting a lack of awareness of antibiotic resistance in *A. baumannii* during this period. Therefore, we calculated the pooled prevalence based on the data during 2006–2016. The pooled prevalence of resistance for each antibiotic was calculated after fitting the random-effect regression, displayed in forest plots (Supplementary Figure S3–12) and summarized in Table 2. The pooled prevalence of antibiotic resistance during 2006–2016 in non-OECD countries is much higher than that of OECD countries for nearly all the studied antibiotics except colistin and tigecycline, the resistance to which are comparable (Table 2). In the case of imipenem, the pooled prevalence of resistance in OECD countries is 53.8% (95% CI 36.8–70.8), in contrast to 76.8% (67.4–86.2) in non-OECD countries. A similar observation was noted on other antibiotics, among which meropenem and amikacin in non-OECD countries have the largest difference in the pooled prevalence of resistance than those in OECD countries. The pooled prevalence of resistance to cefepime is higher but less dramatic in non-OECD countries compared to that in OECD countries. On the other hand, the antibiotic resistance in different countries within OECD varies significantly. As shown in Fig. 2, French Guiana has the lowest pooled prevalence of imipenem resistance at 7%, whereas Italy and Romania at 87 and 95%, respectively. Similar results were also observed on other antibiotics (Supplementary Figure S4–10).

**Table 2:** The pooled prevalence of antibiotic resistance to the commonly prescribed antibiotics in OECD and non-OECD countries during 2006–2016

	OECD				Non-OECD			
Antibiotic	Pooled prevalence (%)	No of isolates tested	No of reporting studies (countries)	*I*^2^ (%)	Pooled prevalence (%)	No of isolates tested	No of reporting studies (countries)	*I*^2^ (%)
Imipenem	53.8 (36.8–70.8)	26,376	22 (11)	64.3	76.8 (67.4–86.2)	7085	18 (10)	44.5
Amikacin	44.6 (24.9–64.3)	25,988	18(10)	60.8	75.9 (66.1–85.8)	7113	18 (10)	46.0
AMP-SUL	47.6 (24.7–70.6)	17,908	12 (7)	60.8	68.1 (61.9–74.3)	3172	7 (4)	15.1
Tobramycin	48.1 (23.1–89.9)	23,064	8 (4)	59.2	67.9 (43.7–92.0)	774	6 (4)	57.1
Ceftazidime	75.2 (60.8–90.3)	5370	19 (11)	62.4	91.5 (84.3–98.6)	7055	17 (11)	28.1
Meropenem	55.7 (38.5–72.9)	23,106	26 (10)	60.4	82.7 (71.6–93.9)	6825	16 (11)	56.7
PIP-TAZ	64.4 (43.6–85.3)	4393	19 (10)	64.6	84.6 (73.8–95.3)	6423	12 (8)	40.6
Cefepime	65.4 (47.6–83.1)	6175	16 (11)	62.7	88.2 (78.9–97.5)	6804	14 (9)	45.6
Colistin	1.4 (0.2–2.6)	5867	15 (11)	18.6	1.3 (0.1–2.7)	1587	13 (9)	16.9
Tigecycline	14.4 (3.6–25.4)	2601	7 (7)	46.7	15.0 (2.0–28.0)	1059	9 (7)	50.7
MDR	56.9 (27.3–86.5)	24,215	8 (6)	63.1	80.4 (69.9–90.8)	508	4 (4)	33.8

*AMP-SUL* ampicillin-sulbactam, *PIP-TAZ* piperacillin-tazobactam

Tigecycline is one of the “last-resort” antimicrobial agents for antibiotic resistance in *A. baumannii* infection. Though it has been used for only about 10 years, significant percentage of resistance has been observed in both OECD and non-OECD countries without significant difference between them (Table 2). Colistin, another last resort of treatment for drug-resistant *A. baumannii* infection, becomes especially important when tigecycline is found to be insusceptible^17,18^. Our analysis showed that the pooled prevalence of colistin resistance were lower at 1.4% (0.2–2.6) and 1.3% (0.1–2.7) in OECD and non-OECD countries during 2006–2016, respectively, suggesting that colistin is still the most active agent against *A. baumannii* infections and potentially effective in clinical practice, although its clinical effectiveness needs more investigations.

### The developmental trend of antibiotic resistance

**Fig. 3:**
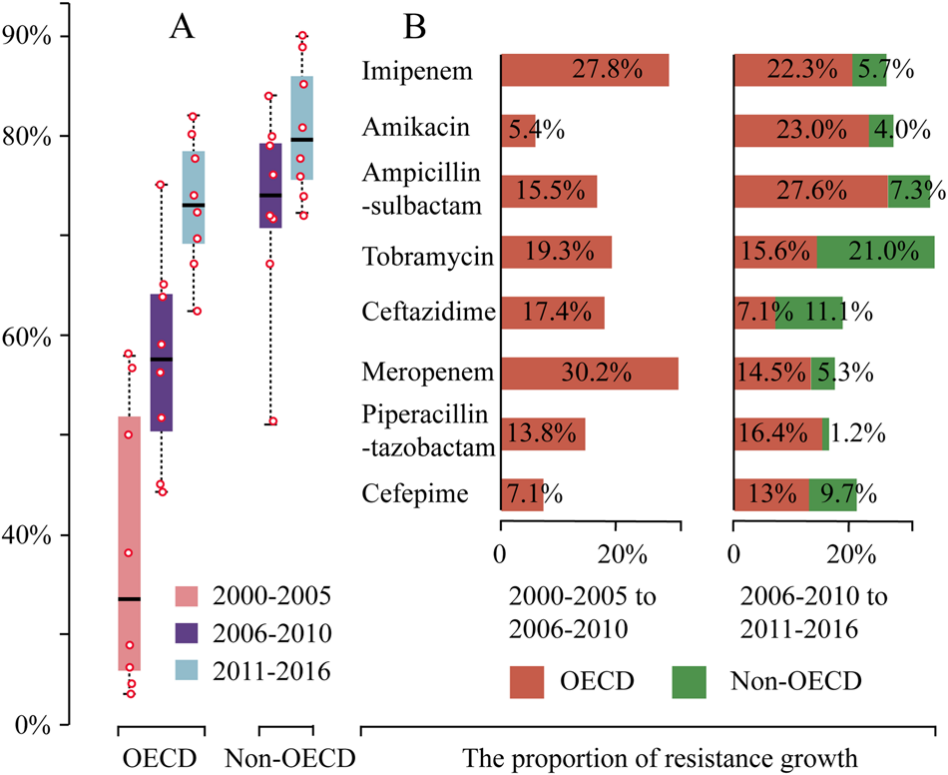
**a** The pooled prevalence of antibiotic resistance to the common antibiotics except for tigecycline and colistin during the three periods (years of 2000–2005, 2006–2010, and 2011–2016) in OECD and two periods (years of 2006–2010 and 2011–2016) in non-OECD countries. **b** The proportion of antibiotic resistance growth between different time period in OECD and non-OECD countries

To get further insight of antibiotic resistance, we analyzed the resistance data by a time scale (Table 3). We included data for the duration of 2000–2005 in OECD countries so to get more information about the resistance development. In contrast to the aforementioned observation that non-OECD countries have significant higher prevalence of antibiotic resistance (Table 2), the pooled prevalence of resistance to each antibiotic during 2011–2016 was comparable between OECD and non-OECD countries, though it was slightly higher in non-OECD countries (Table 3; Fig. 3a). The gap of resistance between OECD and non-OECD countries was getting smaller during past 11 years except for tobramycin and ceftazidime (Supplementary Figure S13). For example, the overall pooled prevalence of resistance to imipenem (2006–2016) in OECD is 23% lower than that in non-OECD countries (Table 2). However, the difference between them becomes negligible during 2011–2016, with 77.8% (66.7–88.4) in non-OECD and 73.9% (54.8–95.6) in OECD countries (Fig. 2; Table 3). The increase in the prevalence of antibiotic resistance in OECD countries was shown to be overall faster than non-OECD countries in the past 11 years (Fig. 3a): For example, the pooled resistance for imipenem increased 22.3% (from 51.6% in 2006–2010 to 73.9% in 2011–2016) in OECD countries, in contrast to 5.7% (from 72.1 to 77.8%) in non-OECD countries (Table 3; Fig. 3b).

**Table 3:** The pooled prevalence of antibiotic resistance to the commonly prescribed antibiotics at three different periods in OECD and non-OECD countries

	OECD (95% CI)			Non-OECD (95% CI)	
Antibiotic	2000–2005	2006–2010	2011–2016	2006–2010	2011–2016
Imipenem	23.8 (9.7–38.0)	51.6 (34.4–68.9)	73.9 (54.8–95.6)	72.1 (58.2–86.0)	77.8 (66.7–88.4)
Amikacin	38.2 (18.0–58.8)	43.6 (21.9–65.3)	66.6 (40.4–92.7)	72.2 (61.6–82.7)	76.2 (59.0–93.4)
AMP-SUL	29.2 (5.6–52.8)	44.7 (23.1–66.1)	72.3 (48.9–95.8)	66.7 (63.7–69.8)	74.0 (58.9–88.1)
Tobramycin	27.4 (13.6–41.1)	46.7 (21.6–71.8)	62.3 (37.1–87.5)	51.4 (41.0–58.9)	72.4 (49.8–95.0)
Ceftazidime	57.3 (37.5–77.2)	74.7 (55.6–93.8)	81.8 (65.4–95.5)	78.6 (66.0–91.0)	89.7 (82.2–97.1)
Meropenem	25.4 (10.4–40.2)	55.6 (42.9–68.3)	70.1 (46.5–93.6)	75.8 (58.6–93.1)	81.1 (69.4–92.7)
PIP-TAZ	49.9 (25.7–74.1)	63.7 (36.1–91.4)	80.1 (61.0–100)	84.0 (72.9–95.2)	85.2 (77.0–93.5)
Cefepime	57.9 (46.0–69.8)	65.0 (46.9–82.1)	78.2 (51–100)	79.7 (63.8–95.6)	89.4 (81.4–97.6)
Colistin	NA	0.0 (0.0–1.0)	1.3 (0.0–2.9)	0.0 (0.0–1.0)	1.6 (0.0–3.7)
Tigecycline	NA	11.3 (3.1–19.4)	13.1 (3.5–22.7)	14.0 (3.9–25.1)	14.1 (2.3–25.8)

*AMP-SUL* ampicillin-sulbactam, *PIP-TAZ* piperacillin-tazobactam, *NA* not avalibale

In addition, our analysis demonstrated that *A. baumannii* has a high propensity to develop resistance rapidly (Fig. 3). In the case of imipenem, the pooled resistance is only 23.8% in OECD countries during 2000–2005, it rapidly increased 50.1 up to 73.9% (54.8–95.6) during 2011–2016 after about 11 years’ time usage only. Similar results were also observed for other antibiotics (Table 3).

### The pooled prevalence of MDR

**Fig. 4:**
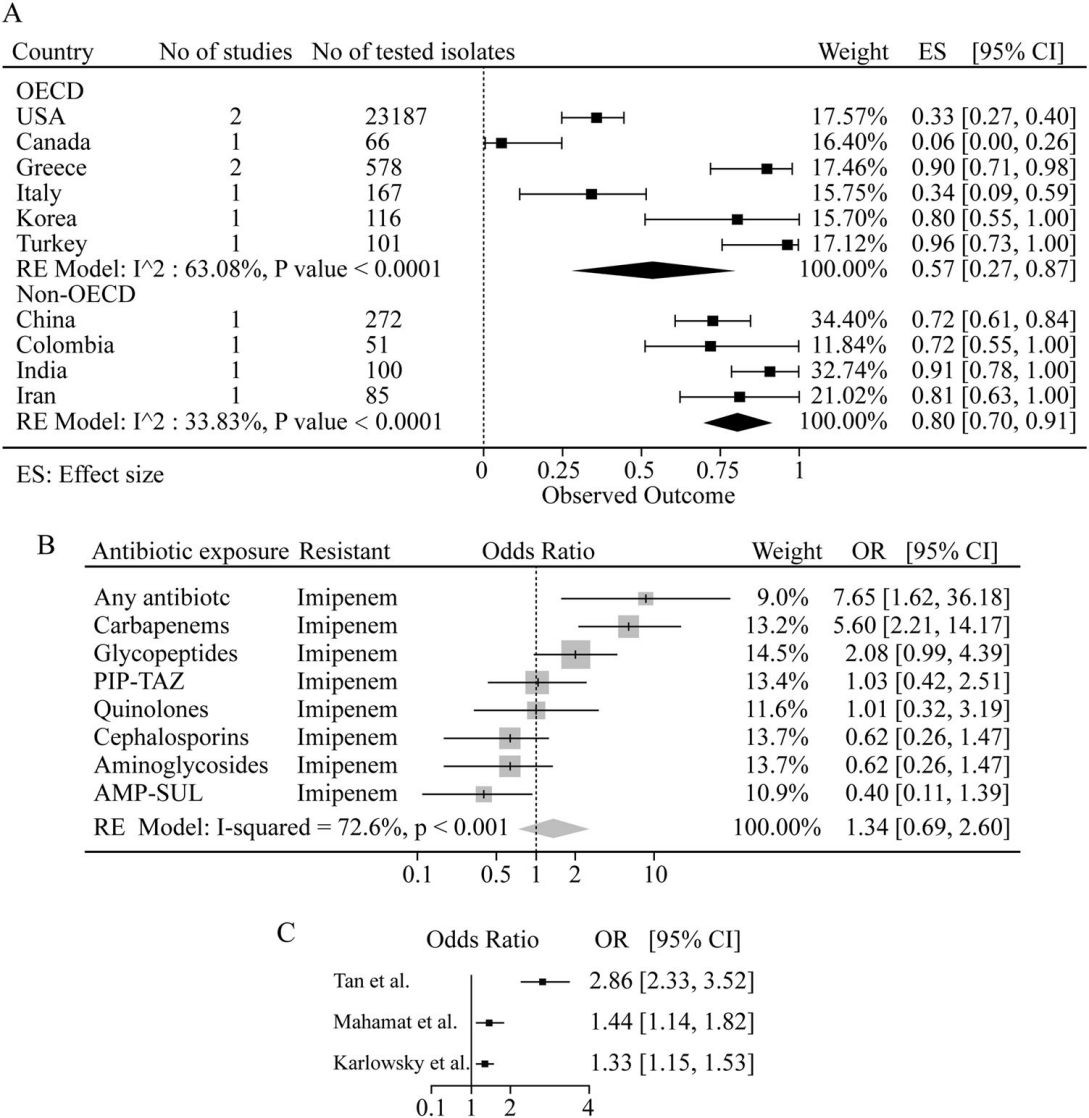
**a** The pooled prevalence of MDR in *A. baumannii* isolates from 2006–2016 in OECD and non-OECD countries. **b** The pooled crude odds ratios between antibiotic resistance in *A. baumannii* infections and prior exposure to any antibiotic. **c** The pooled crude odds ratios between antibiotic resistance in *A. baumannii* infections and ICU or non-ICU hospitalization. *PIP-TAZ* piperacillin-tazobactam, *AMP-SUL* ampicillin-sulbactam

We further analyzed the MDR isolates during 2006–2016. Our analysis disclosed a high MDR prevalence in both OECD and non-OECD countries, with 56.9% (27.3–86.5) and 80.4% (69.9–90.8) respectively (Fig. 4a). The prevalence of MDR in Greece and Turkey were the highest at 90% (81–98) and 96% (83–100) respectively, in contrast to 6% (0–26) in Canada, indicating a significantly different status of MDR within OECD countries. This difference was less significant within non-OECD countries (Fig. 4a).

MDR *A. baumannii* infections are often associated with high mortality^19^. Our analysis showed that the pooled mortality of MDR can reach up to 42.7% (36.7–48.7) worldwide (Supplementary Figure S14) and varies significantly in different countries. For example, America has the lowest pooled mortality of MDR at 29%, whereas Turkey and Korea have nearly twice of that at 64 and 52%, respectively.

### The risk factors for antibiotic resistance

We also investigated the relationship between previous antibiotics exposure and imipenem resistance. As shown in Fig. 4b, an odds ratio of imipenem resistance at 7.65 (1.62–36.18) was observed with patients previously exposed to any antibiotic. Especially, previous exposure to carbapenems (meropenem and imipenem) and glycopeptides resulted in a significant resistance to imipenem, with odds ratios at 5.60 (2.21–14.17) and 2.08 (0.99–4.39), respectively, suggesting that the previous exposure to carbapenems and glycopeptides is the risk factor for imipenem resistance.

A meta-regression analysis on the data from three early studies^20–22^ showed higher odds ratios of antibiotic resistance in patients from intensive care unit (ICU) compared to non-ICU (Fig. 4c). Especially, antibiotic resistance reported by Tan and colleagues demonstrated the highest odds ratio of 2.86 (2.33–3.52) in ICU compared to non-ICU^21^.

## Discussion

To our knowledge, we provide the first systematic review and meta-analysis on the global prevalence of antibiotic resistance in *A. baumannii* infection on the commonly prescribed antibiotics. Our initial analysis found that OECD countries have substantially lower pooled prevalence of antibiotic resistance (2006–2016), which is consistent with our initial expectation as OECD countries have a better antibiotics management system. However, a different conclusion was drawn when we analyzed the prevalence following a time scale. We found that the prevalence of resistance was increasing rapidly in the last 16 years (OECD countries) or 11 years (non-OECD countries). Strikingly, the prevalence increase in OECD countries was even faster and outstanding, which resulted in a similar level of antibiotic resistance to non-OECD countries during 2011–2016 (Fig. 3). The pooled prevalence during 2011–2016 should maximally reflect the current status of antibiotic resistance. Thus, we concluded that the current prevalence of antibiotic resistance in *A. baumannii* infection is similar between OECD and non-OECD countries, both of which are facing the same degree of severity of antibiotic resistance. In addition, our analysis demonstrated how rapid the antibiotic resistance to *A. baumannii* infections expanded, providing a theoretical basis for the proper estimation of the future scenario, which has been challenged for the accuracy in the government report due to the lack of scientific evidence^1,2^.

The rapid expansion of antibiotic resistance discovered in OECD countries could be mainly due to the spread of already established clones or because of acquisition of resistance determinants by susceptible strains. Despite it is not clear which is the main cause, several researches supported the latter^23–27^. Indeed, the pan-European epidemic clonal I, II and III have been universally identified throughout many countries in Europe^28^. However, not every country in Europe has high prevalence of antibiotic resistance. For example, Turkey and Greece have particularly high antibiotic resistance rates. In contrast, Croatia and Poland have an overall much lower resistant rate (Supplementary Figure S3–10). Thus, it is unlike that the rapid increase of antibiotic resistance was only caused by the spread of certain clonal type. The observation could be the combination of several factors: the influence of cross-border exchange, such as transfer of patients, traveler, medical tourism and refugees from the regions, where antibiotic resistance is higher, including Iran and Iraq;^29^ Overuse and/or unrestricted availability of antibiotic agents such as carbapenems;^30^ The difference in antibiotics management and infection control measures, and the environmental pollution^31^.

The high global prevalence of antibiotic resistance found in our studies indicates that most of the antibiotics are likely ineffective to *A. baumannii* infection except tigecyline and colistin, the resistance is much lower (Table 2). Thus, the use of the antibiotics should be cautious, as they might not be effective for the patients. A proper bacterial susceptibility test should be given to understand the resistance profile if antibiotic resistance was suspected. Especially when the patients have previous exposed to antibiotics or hospitalized in ICU. Based on our analysis, susceptibility test to imipenem might be particularly necessary if the patient has been treated with carbapenem and glycopetides previously. On the other hand, continuous surveillance of *A. baumannii* resistance should be implemented to understand the local antibiotic resistance situation and guide the therapeutic practice. Strict infection control policy and antibiotic management should also be enforced to reduce the emergence and spread of pan-antibiotic resistance.

MDR has always been the most serious concern as it can lead to the failure of antimicrobial therapy and increased mortality^19^. We showed that both OECD and non-OECD countries have high MDR rates. On the other hand, the pipeline of new antibiotics for treatment is running dry. The only option we have now is tigecycline which was licensed in 2005, and the previous abandoned antibiotic colistin^32^. Our analyses showed that tigecycline and colistin have the least drug resistance currently (Table 3). Both of them could be potentially used for therapeutic purpose on MDR *A. baumanii* infection. However, the effectiveness of tigecycline was challenged in a recent study^18^. Colistin has a good susceptibility compared to other tested antibiotics. This is likely due to the fact that colistin has been limited from being used during the last several decades due to nephrotoxicity. Now, colistin has been revaluated for treatment of MDR *A. baumannii* for its low resistance^33^. However, resistance to colistin is also emerging. A recent study from America reported 50% resistance to colistin^34^. Most importantly, our analysis found that the development of drug resistance in *A. baumannii* is fast (Fig. 3). It took only 11 years for the percentage of resistance to imipenem increased from 23.8 to 73.9% in OECD countries (Table 3). Thus, we would expect that resistance to tigecycline and colistin could be common in another ten to 20 years’ time if no preventative measures were taken, which may result in an absolute “post-antibiotic era” for pan-antibiotic resistant *A. baumannii* infections. The public awareness of such severity of pan-antibiotic resistance in *A. baumannnii* infections should be increased and the investment by pharmaceutical companies and governments on the new antibiotic research and development should be encouraged.

We noticed that different methods were used for *A. baumannii* identification and antibiotics susceptibility testing in the reports used for our systematic review and meta-analysis, which could be the limitation of our study.

In conclusion, the antibiotic resistance in *A. baumannii* infection has become a global crisis. OECD countries have demonstrated a faster increase in antibiotics resistance in the past 16 years and reached a comparable antibiotic resistance prevalence with non-OECD countries currently. The fast development of antibiotic resistance in *A. baumannii* infection warn of an absolute “post-antibiotic era” for antibiotic resistant *A. baumannii* infections if resistance to tigecycline and colistin become common in the next 10–20 years. Thus, immediate actions should be taken to prevent and control further development of antibiotics resistance in *A. baumannii* infection. Research and development on new antibiotics for *A. baumannnii* infection should be highly encouraged.

## Electronic supplementary material

Supplementary materials
