# Supplementary material for: Analysis of global prevalence of antibiotic resistance in Acinetobacter baumannii infections disclosed a faster increase in OECD countries
Source: Emerg Microbes Infect. 2018 Mar 14;7:31. doi: 10.1038/s41426-018-0038-9 (PMC5849731; doi:10.1038/s41426-018-0038-9)
Supplement: Supplementary file 1 — Supplementary materials [file 41426_2018_38_MOESM1_ESM.doc]

**Analysis of Global Prevalence of Antibiotic Resistance in *Acinetobacter baumannii* Infections Disclosed a Faster Increase in OECD Countries**

Ruiqiang Xie^1^, Xiaohua Douglas Zhang^1^, Qi Zhao^1^, Bo Peng^2^, Jun Zheng^1*^

^1^ Faculty of Health Sciences, University of Macau, Macau SAR, China;

^2^ School of Life Sciences, Sun Yat-sen University , Guangzhou 510006, China; Laboratory for Marine Biology and Biotechnology, Qingdao National Laboratory for Marine Science and Technology，Qingdao 266071, China

^*^ Corresponding author. Email: [Junzheng@umac.mo](mailto:JunZheng@umac.mo)

Zip code: 000000

Running title: Meta-analysis on *Acinetobacter baumannii* infections

Key words: *Acinetobacter baumannii*; antibiotic resistance; systematic review; meta-analysis.

**SUPPLEMENTARY INFORMATION**

1. **Supplementary Table S1**: The Medline and Embase search strategy
2. **Supplementary Table S2**: The characteristics of the 54 studies in the meta-analysis ^1-54^
3. **Supplementary Table S3**: The antimicrobial susceptibility testing method and breakpoints for tigecycline
4. **Supplementary Table S4**: The antimicrobial susceptibility testing method and breakpoints for colistin
5. **Supplementary Figure S1**: The pooled prevalence of ciprofloxacin resistance in *A. baumannii* isolates from patients during 2006-2016, stratified by OECD status
6. **Supplementary Figure S2**: The quality of studies assessed by Cochrane collaboration risk of bias tool
7. **Supplementary Figure S3-12**: The pooled prevalence of resistance for each antibiotic in *A. baumannii* isolates from patients during 2006-2016, stratified by OECD status
8. **Supplementary Figure S13:** The gap of resistance between OECD and non-OECD countries for each antibiotic during 2006-2010 and 2011-2016
9. **Supplementary Figure S14**: The pooled mortality of MDR *A. baumannii* infections worldwide during 2006-2016^13, 25, 63-83^
10. **Supplementary Reference**

**Supplementary Table S1: The Medline and Embase search strategy**

| The MEDLINE and EMBASE search strategy |  |
| --- | --- |
| 1. Exp. Drug Resistance, Microbial | 16. nosocomial.mp |
| 2. Anti-bacterial Agents/ Therapeutic use | 17. Intensive care unit?.tw |
| 3. Antibiotic$.tw | 18. ICU?.tw |
| 4. Antimicrobial$.tw | 19. 10 or 11 or 12 or 13 or 14 of 15 or 16 or 17 or 18 |
| 5. Antimicrobial resistance.mp | 20. Exp. A* baumannii Infections/ Diagnosis |
| 6. Bacterial resistance.mp | 21. Exp. A* baumannii Infections/ Epidemiology |
| 7. Antibiotic resistance.mp | 22. Exp. A* baumannii Infections/ Prevention and Control |
| 8. resistan$.tw | 23. Exp. A* baumannii Infections/ Transmission |
| 9. 1 or 2 or 3 or 4 or 5 or 6 or 7 or 8 | 24. Exp. A* baumannii Infections/ Microbiology |
| 10. Exp. hospital infections | 25. A* baumannii Infections/ Epidemiology |
| 11. Exp. nosocomial Infections/ Microbiology | 26. Acinetobacter baumannii infection.mp |
| 12. Exp. nosocomial Infections/ Transmission | 27. Acinetobacter baumannii.ab.ti |
| 13. Exp. nosocomial Infections/ Epidemiology | 28. A* baumannii isolate$.tw |
| 14. outpatient$.tw or inpatient$.tw | 29. 20 or 21 or 22 or 23 or 24 or 25 or 26 or 27 or 28 |
| 15. hospital.tw | 30. 9 and 19 and 29 |

**Supplementary Table S2: The characteristics of the 54 studies in the meta-analysis ^1-54^**

| Ref. No. | First Author | Country | Design | n | Species identification | Antibiotic  susceptibilities | Antimicrobial susceptibility testing | Breakpoints |
| --- | --- | --- | --- | --- | --- | --- | --- | --- |
| OECD | | | | | | | | |
| 1 | Koeleman (2001) | Netherlands | RO | 23 | Amplified fragment length polymorphism (AFLP) ^55^ | AMI; GEN; TOB; CIP; TSU; AMP; ACA; PIP-TAZ; CFU; CTX; CFZ; IMI; MER | Vitek System | NCCLS |
| 2 | Sahm (2001) | USA | PO | 5775 | Collected from 27 hospital laboratories and requested to provide defined quotas of a specific species | AMP; CFZ; CFX; IMI; PIP-TAZ; GEN; CIP; LEV; SXT | Broth microdilution | NCCLS |
| 3 | Martin-Lozano (2002) | Spain | CC | 109 | MicroScan, the API 20NE System and the temperature growth test (44°C) | AMP; AMP-SUL; AZT; IMI; CTX; CFZ; GEN; AMI; TET; CIP; SXT; COL | MicroScan system and Etest | NCCLS |
| 4 | Karlowsky (2003) | USA | RO | 5636 | The Surveillance Network Database-USA ^56^ | AMI; CF; CFZ; CIP; GEN; IMI; LEV; MER; PIP-TAZ; TIC-clavulanate | Standard method according to U.S. FDA and NCCLS | U.S. FDA and NCCLS |
| 5 | Katragkou (2006) | Greece | CC | 84 | Vitek-2 automated system | IMI; CFZ; AMI; GEN; NET; TOB; AMP; PIP-TAZ; TIC; CIP; ACA; TIC-clavulanic acid | Vitek-2 automated system | Vitek-2 automated system |
| 6 | Arda (2007) | Turkey | PO | 112 | Conventional methods and API systems | CFX; CFP; CF; PIP-TAZ; NET; LEV; CIP; IMI; MER | Disk diffusion | NCCLS |
| 7 | Ko (2007) | Korea | RO | 214 | rpoB gene analysis ^57^ | COL; TET; CIP; AMI; MER; IMI; CF; CFX; CFZ; PIP-TAZ; AMP-SUL; Polymyxin B | Broth microdilution | CLSI 2006 |
| 8 | Landman (2007) | USA | RO | 1286 | Identified by the participating laboratories according to standard techniques ^58, 59^ | AMP-SUL; PIP-TAZ; CFZ; CF; IMI; MER; AMI; CIP; TIG; Polymyxin B | Susceptibility testing according to CLSI standards | CLSI 2005 and MICs≤2 mg/L were considered susceptible to tigecycline (U.S. FDA) |
| 9 | Oteo (2007) | Spain | PO | 354 | Determined by the Phoenix System, the API 20 NE method, the standard phenotypic reference methods, including the growth at 44 °C, and amplified ribosomal DNA restriction analysis (ARDRA) | AMP; ACA; AMP-SUL; PIP-TAZ; CFZ; CF; IMI; MER; GEN; TOB; AMI; CIP; SXT | Broth microdilution and Etest | CLSI 2005 |
| 10 | Baran (2008) | Turkey | CC | 123 | VITEK automated system | AMI; AZT; CF; CFZ; CIP; GEN; NET; PIP; PIP-TAZ; TIC; TOB; SXT; Pefloxacin; TIC-clavulanate | VITEK | VITEK |
| 11 | Dauner (2008) | USA | CS | 129 | Identified in PathNet (Cerner, Kansas City, MO, USA) | AMI; AMP-SUL; CF; CFZ; CIP; GEN; IMI; LEV; MER; TET; TOB; SXT; TIC-clavulanate | Broth microdilution | NR |
| 12 | Gbaguidi-Haore (2008) | France | RO | 688 | API 20NE system | PIP-TAZ; CFZ; CF; IMI; CIP; AMI; TOB; TCA | disk diffusion | CA-SFM |
| 13 | Rodriguez (2008) | Spain | RO | 63 | The identification of *A. baumannii* was based on its growth at 37°C, 41°C and 44°C, negative oxidase and catalase reactions, motility testing and production of acid from glucose, and it was confirmed using a commercial identification system | AMP-SUL; PIP-TAZ; TIC; CTX; CFZ; CF; IMI; MER; GEN; TOB; AMI; CIP; SXT; AZT; TET | Broth microdilution | CLSI 2007 |
| 14 | Van (2009) | Netherlands | PO | 88 | Vitek2 system and AFLP analysis | CFZ; PIP; MER; IMI; GEN; NET; TOB; AMI; OFL; AMO-SUL; TET; COT | Disk diffusion | CLSI |
| 15 | Wadl (2010) | Germany | RO | 1190 | Surveillance data from the GENARS-project | CF; CFZ; CIP; PIP-TAZ; TOB; LEV; COT; AMP-SUL; IMI; MER; GEN | Broth microdilution | DIN |
| 16 | McCracken (2011) | Canada | RO | 66 | CANWARD surveillance study | CF; CFX; MER; PIP-TAZ; CIP; LEV; AMI; GEN; COL; TIG; SXT | Broth microdilution | CLSI 2010 |
| 17 | Maraki (2012) | Greece | RO | 137 | Standard biochemical methods, the API system and the Vitek2 automated system | PIP-TAZ; CFX; CTX; CFZ; CF; AMI; MER; TOB; AMI; GEN; NET; TET; CIP; COL; TCA | Vitek2 | CLSI 2010 |
| 18 | Park (2012) | Korea | RO | 287 | VITEK2 system and partial rpoB gene sequences analysis | IMI; MER; COL; TET; CIP; AMI; CF; CFX; CFZ; PIP-TAZ; AMP-SUL; TIG; Polymyxin B; RIF; MDR | Broth microdilution | CLSI 2009 and U.S. FDA |
| 19 | Samonis (2012) | Greece | RO | 1242 | Standard biochemical methods, the API 20NE system and Vitek2 automated system | TIC; PIP; PIP-TAZ; CFZ; CF; AZT; IMI; MER; TOB; AMI; GEN; NET; COL; SXT; CIP; TIC-clavulanic acid; Isepamicin; Pefloxacin | Vitek2 | CLSI 2010 |
| 20 | Dally (2013) | Germany | PO | 60 | Matrix-assisted laser desorption ionization–time-of-flight mass spectrometry using a MALDI Bioanalyser, blaOXA-51-like gene detection ^60^ | PIP-TAZ; CFZ; IMI; MER; SXT; AMI; GEN; TOB; CIP; TIG; COL | Vitek2 | CLSI, EUCAST |
| 21 | De (2013) | Italy | RO | 167 | VitekTM system | AMI; CFZ; CIP; IMI; PIP-TAZ | Vitek2 | CLSI 2010 |
| 22 | Metan (2013) | Turkey | RO | 100 | Compared to the AFLP library of the Leiden University Medical Center | GEN; NET; AMI; CFZ; OFL; TET; AMP-SUL; PIP; IMI; MER; COL; SXT; TOB | Disk diffusion | CLSI 2011 |
| 23 | Morfin-Otero (2013) | Mexio | RO | 3680 | Sensititre ARIS® 2X and blaOXA-51 gene detection | AMI; CF; CFZ; CIP; IMI; MER | Sensititre ARIS® 2X | CLSI |
| 24 | Cicek (2014) | Turkey | RO | 101 | VITEK2, REP-PCR analysis, Multiplex PCR for detection of blaOXA genes | AMP-SUL; PIP; PIP-TAZ; CFZ; CF; AMI; GEN; NET; CIP; LEV; TET; TIG; COL; IMI; MER; Cefoparozone-sulbactam; SXT | Vitek2 and confirmed with Etest | CLSI, 2012 |
| 25 | Fitzpatrick (2015) | USA | RO | 116 | Determined by rpoB gene sequence based on NGS sequencing | AMP-SUL; PIP-TAZ; IMI; CF; CIP; AMI; TOB; TIG; COL; MIN; DOX; MDR | Vitek2, Etest, disk diffusion | CLSI 2013, U.S. FDA |
| 26 | Guzek (2015) | Poland | RO | 418 | VITEK2 automated system | CIP; IMI; MER; CFZ; CF; PIP-TAZ; COL; AMP-SUL; SXT; DOX | VITEK2 | CLSI 2010, EUCAST |
| 27 | Kaliterna (2015) | Croatia | RO | 109 | API 20NE, VITEK2, confirmed by the presence of an OXA-51-type β-lactamase ^61^ | PIP-TAZ; CFZ; CF; AMP-SUL; IMI; MER; AMI; GEN; CIP; COL | Disk diffusion, VITEK2 | CLSI 2007, EUCAST |
| 28 | Spiliopoulou (2015) | Greece | RO | 441 | Gram-negative BD BBL Crystal ID system and VITEK2 system | AMP-SUL; PIP-TAZ; CFZ; IMI; MER; AMI; GEN; TOB; NET; CIP; TIG; SXT; MIN | Disk diffusion, VITEK2, Etest | CLSI 2011, U.S. FDA |
| 29 | Lazureanu (2016) | Romania | RO | 37 | Classical bio-chemical methods, Vitek2 | PIP; TIC; CFZ; AMP-SUL; IMI; MER; CF; Penicillin; Quinolones; TIC-tazobactam; TCA; AZT; cefotaxime | Vitek2 | CLSI 2010 |
| 30 | Mahamat (2016) | French Guiana | RO | 441 | VITEK2, API 20NE method, matrix-assisted laser desorption/ionisation time-of-flight mass spectrometry | PIP; PIP-TAZ; TIC; CFZ; CF; IMI; CIP; AMI; SXT; COL; TCA; Fosfomycin | Disk diffusion, Etest | CLSI 2010 |
| 31 | Zilberberg (2016) | USA | RO | 33503 | Standard method from The Surveillance Network database | AMI; TOB; TET; IMI; MER; AMP-SUL; COL; DOX; MIN; DOR; Polymyxin B; MDR | Standard U.S. FDA-approved testing methods | CLSI 2012 |
| 32 | Mezzatesta (2012) | Italy | RO | 202 | Vitek2, API 20NE system, PFGE and MLST | MER; IMI; PIP; AZT; CFZ; CF; CIP; AMI; GEN; COL; TIG; PIP-TAZ | Broth microdilution | CLSI 2010 |
| 33 | Principe (2014) | Italy | CS | 246 | Phoenix automated microbiology system, Vitek2 system, matrix-assisted laser desorption ionization–time of flight mass spectrometry, detection of blaOXA-51-like alleles | IMI; MER; AMI; GEN; COL; LEV; SXT; AMP-SUL; CFZ; CF; TIG; DOR | Phoenix automated microbiology system, Vitek2, Etest | CLSI 2014, EUCAST |
| 34 | Izdebski (2012) | Poland | RO | 30 | Vitek2, followed by sequencing of the 16S-23S rRNA intergenic spacer | PIP; PIP-TAZ; AMP-SUL; CFZ; CTX; CF; IMI; MER; COL; AMI; GEN; CIP; SXT; TCA | Etest | CLSI 2006 EUCAST |
| 35 | Bonnin (2011) | Romania | RO | 13 | 16S rRNA gene sequencing | TIC; PIP; PIP-TAZ; CL; CFU; CX; CTX; CFZ; CF; MER; IMI; TIC-tazobactam; Cefpirome; DOR | Disk diffusion, Etest | CLSI 2010 |
| Non-OECD | | | | | | | | |
| 36 | Ben (2011) | China Taiwan | RO | 213 | Vitek GNI+ system | AMP; AMI; AMP-SUL; CZ; CF; CFZ; CFX; CIP; GEN; IMI; LEV; PIP-TAZ; SXT | Agar dilution, Etest, Vitek | CLSI |
| 37 | Reguero (2013) | Colombia | RO | 51 | Vitek system, 16S–23S rRNA internal transcribed spacer | CTX; CFZ; CF; PIP-TAZ; AMP-SUL; AZT; IMI; MER; CIP; AMI; GEN | Disk diffusion | CLSI 2011 |
| 38 | Ruan (2013) | China | RO | 2197 | VITEK GNI+ cards, sequence analysis of the 16S–23S rRNA gene intergenic spacer and the intrinsic blaOXA-51-like gene | IMI; MER; CTX; CF; CFZ; PIP; AMI; GEN; CIP; AMP-SUL; PIP-TAZ; MIN | Disk  diffusion | CLSI 2012 |
| 39 | Aly (2014) | Saudi Arabia | RO | 253 | MicroScan, RNA polymerase β-subunit (rpoB) gene PCR and sequencing | AMI; CF; CTX; CFZ; CFX; CIP; COL; GEN; IMI; MER; TOB; SXT | Standard method according to CLSI guideline, Etest | CLSI 2011 |
| 40 | Obeidat (2014) | Jordan | PO | 64 | Presence of specific metallo-b-lactamase genes and OXA-type b-lactamase genes | AMI; CFZ; CIP; COL; IMI; MER; PIP-TAZ; TIG; AZT; GEN | Disk diffusion, Etest | CLSI 2009 |
| 41 | Tan (2014) | China | RO | 3026 | The criteria of the American Society for Microbiology | AMI; SXT; CIP; PIP-TAZ; CFZ; CF; IMI; MER; Cefaperazone-sulbactam | Standard method according to CLSI | CLSI |
| 42 | Bahador (2015) | Iran | RO | 85 | API 20NE System and confirmed by gyrB multiplex PCR | AMI; CFZ; CIP; CFX; COL; CTX; CF; IMI; NET; LEV; PIP; AMP-SUL; SXT; TET; TIG; PIP-TAZ; TOB; DOR; DOX; GEN; MIN; RIF; TCA | Broth microdilution | CLSI 2015 EUCAST |
| 43 | Elabd (2015) | Saudi Arabia | RO | 108 | Conventional biochemical tests, API 20NE and MicroScan Walkaway automated systems | CFZ; CTX; CF; PIP; IMI; MER; CIP; LEV; AMI; TOB; COL; GEN; SXT | MicroScan, Etest | CLSI 2012 |
| 44 | Ku (2015) | China Taiwan | RO | 434 | Identified at the genomic species level by a multiplex PCR assay for the detection of a specific 16S-23S rRNA intergenic spacer | AMP-SUL; AMI; CIP; COL; CF; IMI; LEV; MER; PIP-TAZ; CFZ; TIG; GEN | Agar dilution, Broth microdilution, Etest | CLSI 2012 U.S. FDA |
| 45 | Le (2015) | Vietnam | RO | 74 | API 20NE, PCR method to detect blaOXA-51 | COL; IMI; MER; CFZ; CFX; PIP-TAZ; AMI; COT; CF; LEV; TCA | Disk diffusion, E-test | CLSI 2012 |
| 46 | Lowings (2015) | South Africa | RO | 94 | VITEK2 system, MALDI-TOF MS and OXA-51 gene detection | AMP; CFU; CX; CTX; CFZ; CF; IMI; MER; AMI; GEN; CIP; TIG; NIT; COL; ACA; SXT | Vitek2 | VITEK2 |
| 47 | Modarresi (2015) | Iran | RO | 65 | API 20NE assay and blaOXA-51 gene detection | CXM; GEN; TOB; AMI; PIP; CFZ; CTX; IMI; MER; COL; TIG; RIF | Disk diffusion, Broth microdilution | CLSI 2012, EUCAST |
| 48 | Rynga (2015) | India | RO | 100 | API 20NE strips further confirmed using ARDRA | CFZ; CTX; AZT; IMI; CX; AMI; CIP; GEN; PIP-TAZ; CF; AMP-SUL; TIG; COL; Cefpodoxime | Disk diﬀusion | CLSI 2013 and Jones et al. ^62^ |
| 49 | Shrestha (2015) | Nepal | RO | 122 | Biochemical methods, detection of 16S rRNA, gyrB and bla_OXA-51-l_ike genes, whole-genome sequences | AMI; CFZ; CIP; COL; MER; TIG; Arbekacin | Disk diffusion, Broth microdilution | CLSI 2015 EUCAST and U.S. FDA |
| 50 | Tada (2015) | Vietnam | RO | 93 | Whole genome sequences | IMI; MER; AMI; CIP; COL; TIG | Broth microdilution | CLSI 2015 |
| 51 | Chatterjee (2016) | India | RO | 49 | VITEK2, the Mini API system, ARDRA | CFZ; CF; AZT; IMI; MER; AMI; GEN; CIP; TIG; DOR; MIN | VITEK2, Etest | CLSI 2014 |
| 52 | Mathlouthi (2016) | Libya | RO | 36 | BD Phoenix^TM^ System, matrix-assisted laser desorption/ionisation time-of-flight mass spectrometry method, PCR amplification and sequencing of the intrinsic bla_OXA-51-like_ gene | TIC; PIP-TAZ; CFZ; CTX; CF; AZT; AMI; SXT; TOB; GEN; CIP; MER; IMI; TIG; COL; TCA; SUL; RIF; Ertapenem; MIN | Disk diffusion, Etest | CA-SFM |
| 53 | Pourabbas (2016) | Iran | RO | 59 | The biochemical methods and API 20NE system, gyrB multiplex PCR, partial rpoB gene sequence analysis, multiplex PCR tests | LEV; CIP; IMI; AMI; PIP-TAZ; TIG; AMP-SUL; MER; COL; RIF | Disk diffusion and Etest | CLSI 2014 |
| 54 | Qi (2016) | China | RO | 272 | API 20NE tests | GEN; TOB; AMI; IMI; MER; CIP; LEV; AMP-SUL; PIP-TAZ; CFZ; CFX; CF; SXT; PIP | VITEK2, Broth microdilution, Disk diffusion | CLSI 2015 |

**Note. Design**: RO = retrospective observational; PO = prospective observational; CC = case-control; CS = cross-sectional. **Antibiotic susceptibilities**: AMI = amikacin; AMP = ampicillin; AMP-SUL = ampicillin-sulbactam; AZI = azithromycin; AZT = aztreonam; CC = cefaclor; CD = cedadroxil; CF = cefepime; CFP = cefoperazone; CFU = cefuroxime; CFX = ceftriaxone; CFZ = ceftazidime; CHL = chloramphenicol; CIP = ciprofloxacin; CL = cefalothin; CLX = cefalexin; COA = co-amoxiclav; COT = co-trimoxazole; CP = cefprozil; CTX = cefotaxime; CX = cefoxitin; CXM = cefixime; CZ = cefazolin; GEN = gentamicin; IMI = imipenem; LEV = levofloxacin; MER = meropenem; NA = naladixic acid; NET = netilmicin; NIT = nitrofurantoin; NOR = norfloxacin; OFL = ofloxacin; PIP = piperacillin; PIP-TAZ = piperacillin-tazobactam; STR = streptomycin; SUL = sulfamethoxazole; TAZ = tazobactam; TEM = temocillin; TET = tetracycline; TIC = ticarcillin; TRI = trimethoprim; TOB = Tobramycin; ACA = Amoxicillin-clavulanic acid; SXT = trimethoprim-sulfamethoxazole. **Guidelines**: CLSI = The Clinical and Laboratory Standards Institute; BSAC = British Society for Antimicrobial Chemotherapy; NCCLS: The National Committee for Clinical Laboratory Standards; DIN: German Institute for Standardization; CA-SFM: Antibiogram Committee of the French Society for Microbiology; NR: not reported.

**Supplementary Table S3: The antimicrobial susceptibility testing method and breakpoints for tigecycline**

| Ref. No. | First Author | Country | Antimicrobial susceptibility testing | Breakpoints | Resistant n (%) |
| --- | --- | --- | --- | --- | --- |
| 8 | Landman (2007) | USA | Susceptibility testing according to CLSI standards | MICs≤2 mg/L were considered susceptible to tigecycline (U.S. FDA) | 431 (8) |
| 16 | McCracken (2011) | Canada | Broth microdilution | CLSI 2010 | 66 (6.1) |
| 18 | Park (2012) | Korea | Broth microdilution | U.S. FDA | 127 (4.7) |
| 20 | Dally (2013) | Germany | Vitek2 | Automatized Vitek2 system | 60 (0) |
| 24 | Cicek (2014) | Turkey | Vitek2 and confirmed with Etest | Vitek2 | 101 (8) |
| 25 | Fitzpatrick (2015) | USA | Etest | U.S. FDA | 72 (76) |
| 28 | Spiliopoulou (2015) | Greece | Etest | U.S. FDA | 441 (46) |
| 40 | Obeidat (2014) | Jordan | Etest | MICs≤2 mg/L were considered susceptible to tigecycline (U.S. FDA) | 64 (0) |
| 42 | Bahador (2015) | Iran | Broth microdilution | EUCAST criteria | 85 (34) |
| 44 | Ku (2015) | China Taiwan | Etest | U.S. FDA | 434 (17) |
| 46 | Lowings (2015) | South Africa | Vitek2 | NR | 94 (0) |
| 47 | Modarresi (2015) | Iran | Disk diffusion, Broth microdilution | EUCAST criteria | 65 (7) |
| 48 | Rynga (2015) | India | Disk diﬀusion | Jones et al. ^62^ | 100 (42) |
| 49 | Shrestha (2015) | Nepal | Disk diffusion, Broth microdilution | EUCAST and U.S. FDA | 122 (11) |
| 52 | Mathlouthi (2016) | Libya | Disk diffusion | CA-SFM | 36 (0) |
| 53 | Pourabbas (2016) | Iran | Disk diffusion and Etest | NR | 59 (74.5) |

U.S. FDA criteria: The U.S. FDA breakpoints for Enterobacteriaceae.

EUCAST criteria: the criteria of the European Committee on Antimicrobial Susceptibility Testing (EUCAST) for Enterobacteriaceae were used, in which an MIC of <1 µg/mL was defined as susceptible and >2 µg /mL was considered resistant.

NR: not reported.

**Supplementary Table S4: The antimicrobial susceptibility testing method and breakpoints for colistin**

| Ref. No. | First Author | Country | Antimicrobial susceptibility testing | Breakpoints | Resistant n (%) |
| --- | --- | --- | --- | --- | --- |
| 3 | David (2002) | Spain | Etest | NCCLS | 109 (0) |
| 13 | Rodriguez (2008) | Spain | Broth microdilution | CLSI 2007 and BSAC | 63 (0) |
| 16 | McCracken (2011) | Canada | Broth microdilution | CLSI 2010 | 66 (6.1) |
| 17 | Maraki (2012) | Greece | Vitek2 | CLSI 2010 | 137 (5.8) |
| 18 | Park (2012) | Korea | Broth microdilution | CLSI 2009 | 127 (2.4) |
| 19 | Samonis (2012) | Greece | Vitek2 | CLSI 2010 | 1242 (0) |
| 20 | Dally (2013) | Germany | Vitek2 | EUCAST | 60 (0) |
| 22 | Metan (2013) | Turkey | Disk diffusion | BSAC 2007 | 100 (0) |
| 24 | Cicek (2014) | Turkey | Vitek2 and confirmed with Etest | CLSI 2012 | 101 (0) |
| 25 | Fitzpatrick (2015) | USA | Etests | CLSI 2013 | 72 (4) |
| 26 | Guzek (2015) | Poland | Vitek2 | CLSI 2011 | 418 (0) |
| 27 | Kaliterna (2015) | Croatia | Broth microdilution | EUCAST | 109 (0) |
| 30 | Mahamat (2016) | French Guiana | Disk diffusion | CLSI 2010 | 441 (3.4) |
| 31 | Zilberberg (2016) | USA | Antimicrobial susceptibility testing according standard U.S. FDA–approved testing methods | CLSI 2012 | 2086 (5.3) |
| 32 | Mezzatesta (2012) | Italy | microdilution | CLSI 2010 | 202 (0.4) |
| 33 | Principe (2014) | Italy | Broth microdilution | EUCAST | 246 (0) |
| 34 | Izdebski (2012) | Poland | Etest | EUCAST | 30 (0) |
| 39 | Aly (2014) | Saudi Arabia | Etest | CLSI 2011 | 253 (1.19) |
| 40 | Obeidat (2014) | Jordan | Disk diffusion, Etest | CLSI 2009 | 64 (0) |
| 42 | Bahador (2015) | Iran | Broth microdilution | CLSI 2015 | 85 (12) |
| 43 | Elabd (2015) | Saudi Arabia | MicroScan Walkaway automated systems and Etest | CLSI 2012 | 108 (4.4) |
| 44 | Ku (2015) | China Taiwan | Agar dilution | CLSI 2012 | 434 (0) |
| 45 | Minh (2015) | Vietnam | Etest, Broth microdilution | CLSI 2012 | 74 (0) |
| 46 | Lowings (2015) | South Africa | Vitek2 | NR | 94 (0) |
| 47 | Modarresi (2015) | Iran | Disk diffusion, Broth microdilution | CLSI 2012 | 65 (13) |
| 48 | Rynga (2015) | India | Disk diﬀusion | CLSI 2013 | 100 (3) |
| 49 | Shrestha (2015) | Nepal | Disk diffusion, Broth microdilution | CLSI 2015 | 122 (0) |
| 50 | Tada (2015) | Vietnam | Broth microdilution | CLSI 2015 | 93 (5) |
| 52 | Mathlouthi (2016) | Libya | Disk diffusion | CA-SFM | 36 (0) |
| 53 | Pourabbas (2016) | Iran | Disk diffusion, Etest | CLSI 2014 | 59 (0) |

Note. NCCLS: The National Committee for Clinical Laboratory Standards. BSAC: The British Society for Antimicrobial Chemotherapy (BSAC) criteria. CLSI: The Clinical and Laboratory Standards Institute

**Supplementary Figure S1:** The pooled prevalence of ciprofloxacin resistance in *A. baumannii* isolates from patients during 2006-2016, stratified by OECD status


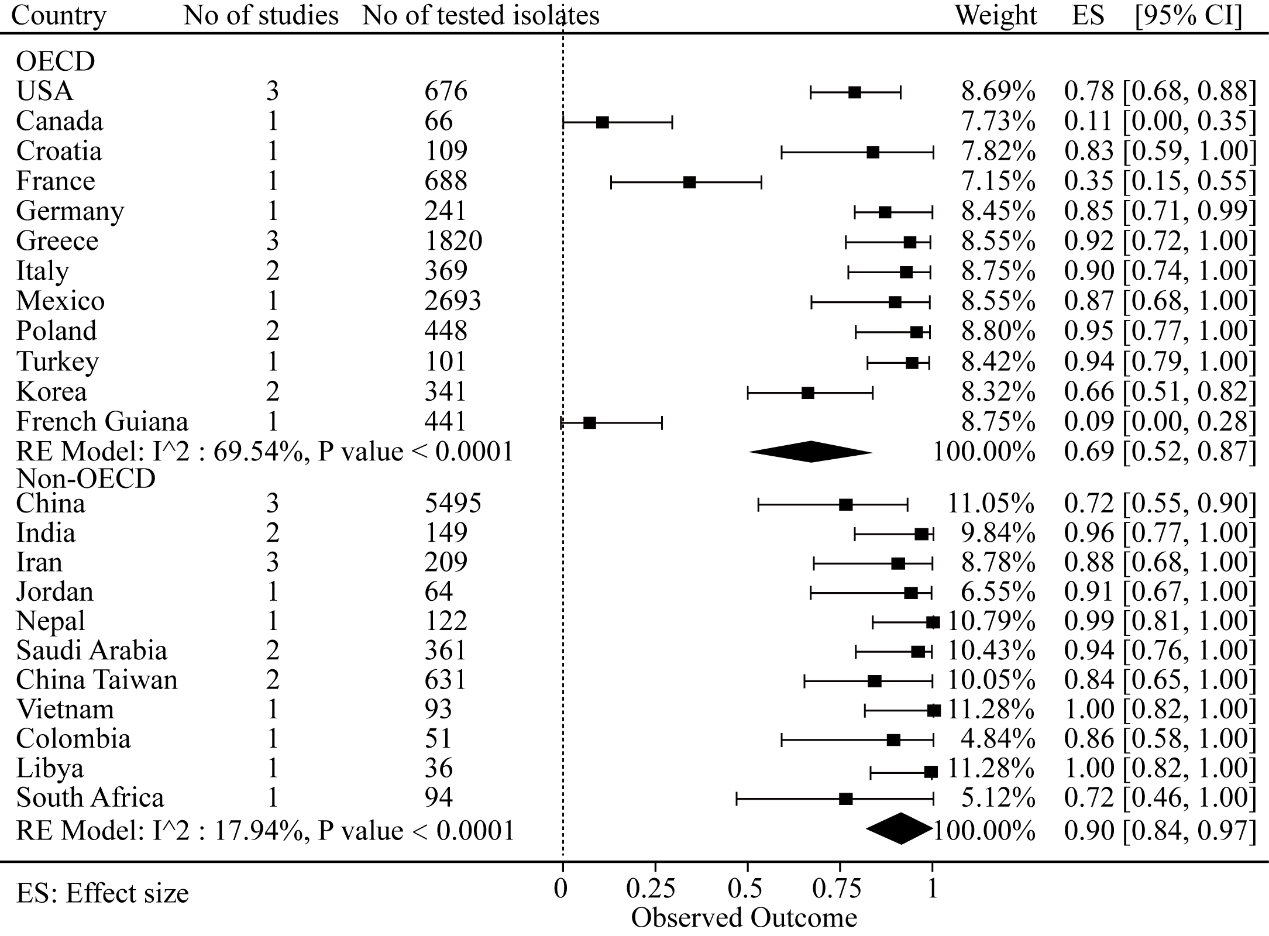


**Supplementary Figure S2:** The quality of studies assessed by Cochrane collaboration risk of bias tool


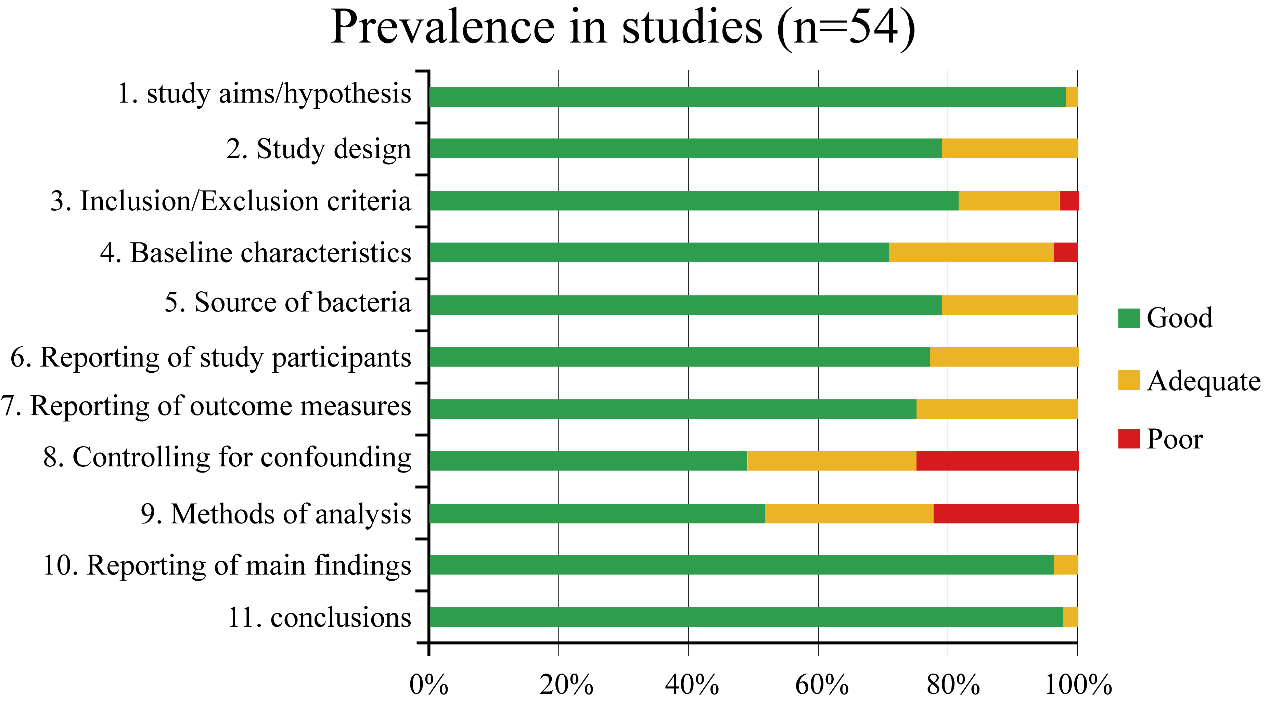


**Supplementary Figure S3:** The pooled prevalence of imipenem resistance in *A. baumannii* isolates from patients during 2006-2016, stratified by OECD status


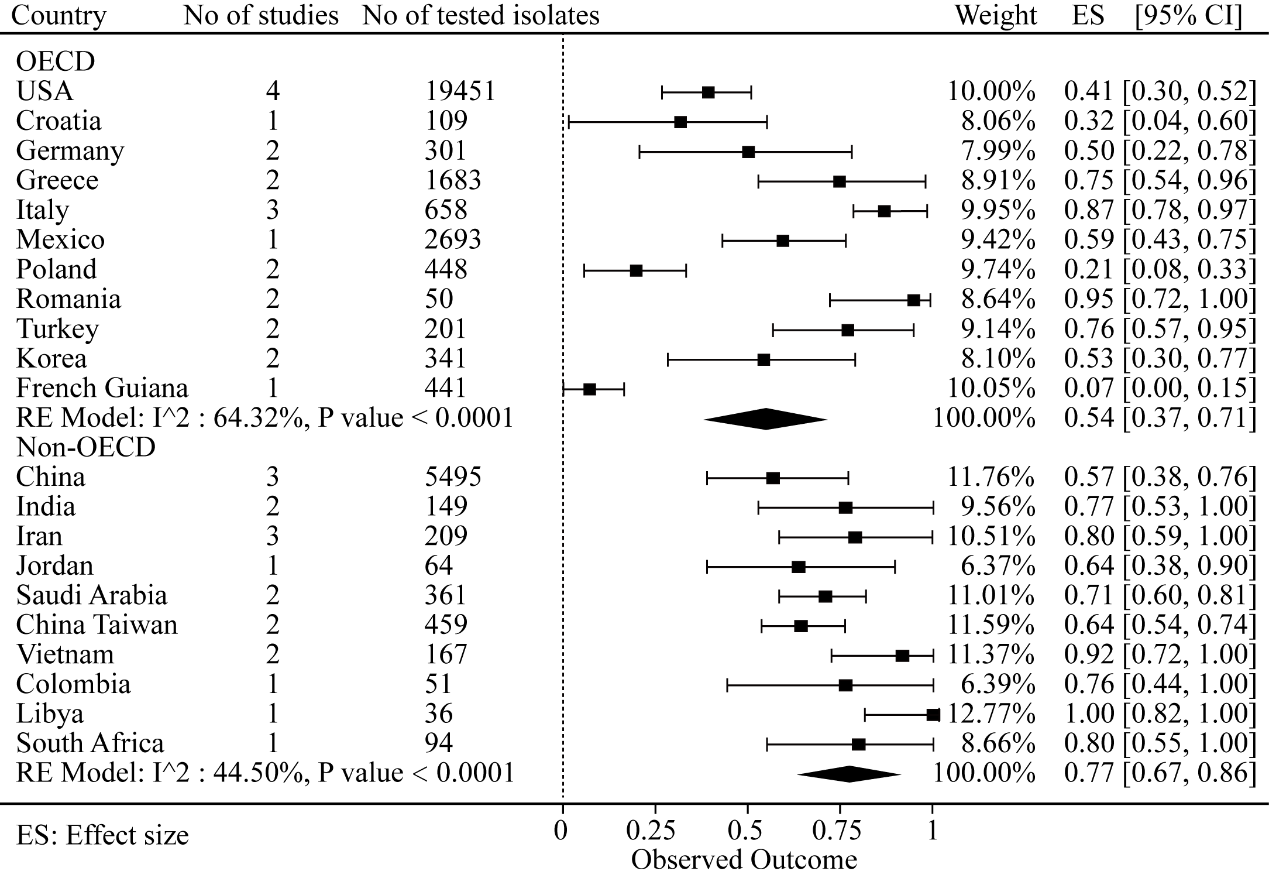


**Supplementary Figure S4:** The pooled prevalence of amikacin resistance in *A. baumannii* isolates from patients during 2006-2016, stratified by OECD status


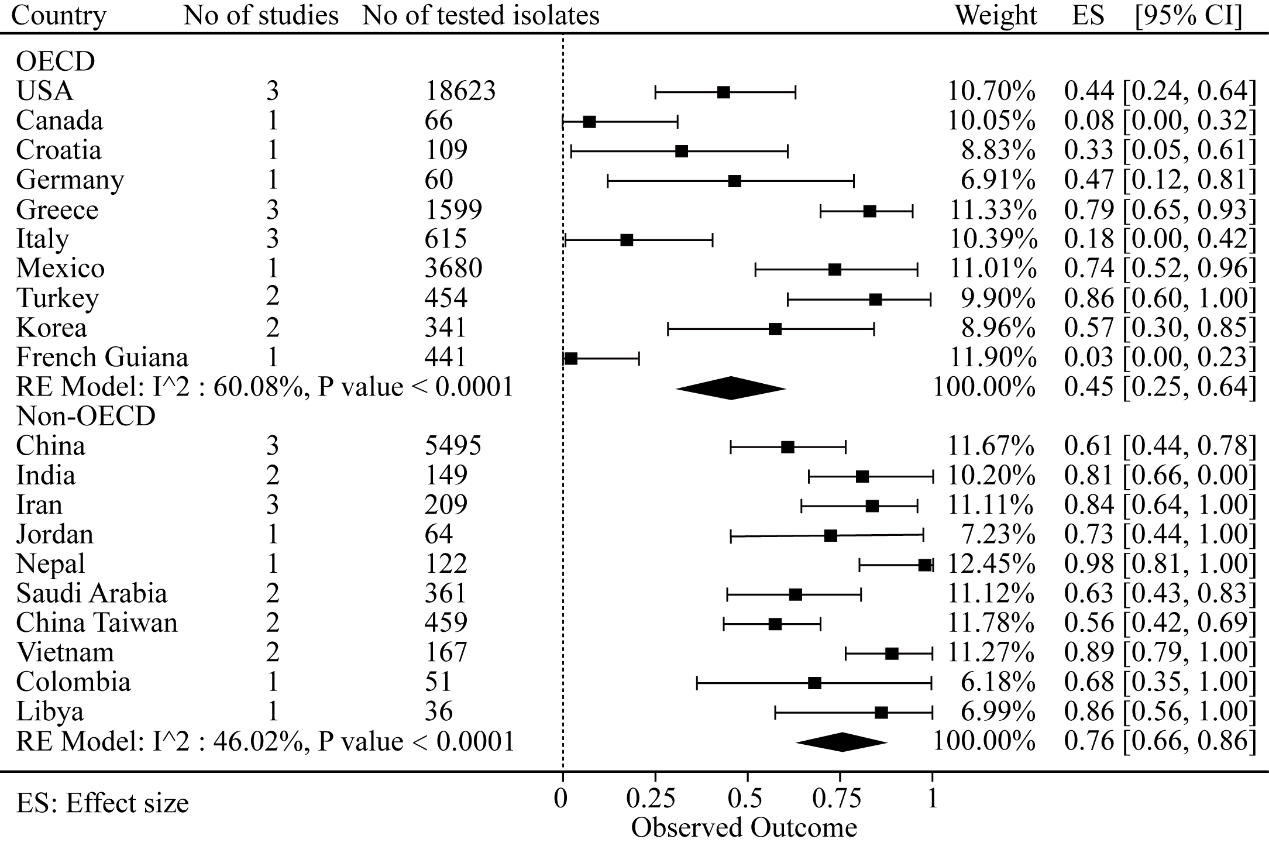


**Supplementary Figure S5:** The pooled prevalence of ampicillin-sulbactam resistance in *A. baumannii* isolates from patients during 2006-2016, stratified by OECD status


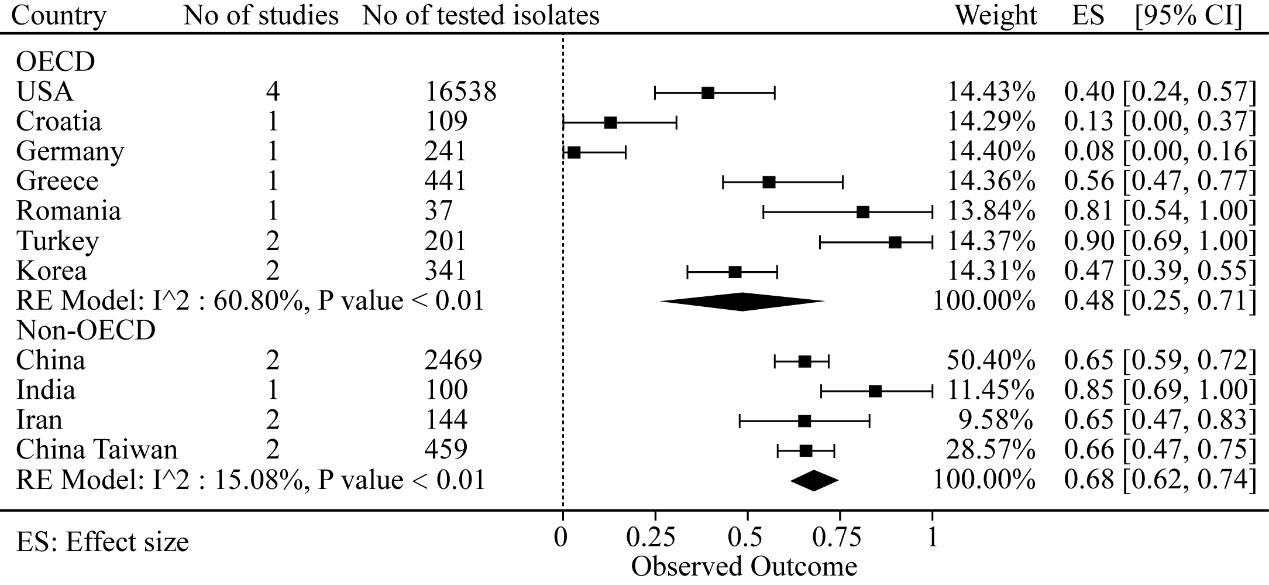


**Supplementary Figure S6:** The pooled prevalence of tobramycin resistance in *A. baumannii* isolates from patients during 2006-2016, stratified by OECD status


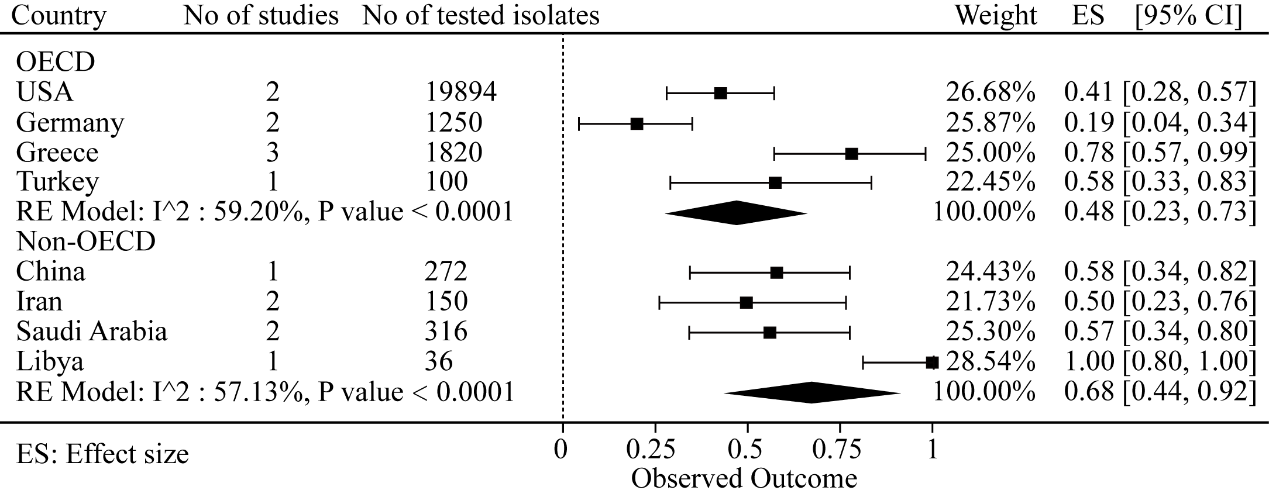


**Supplementary Figure S7:** The pooled prevalence of ceftazidime resistance in *A. baumannii* isolates from patients during 2006-2016, stratified by OECD status


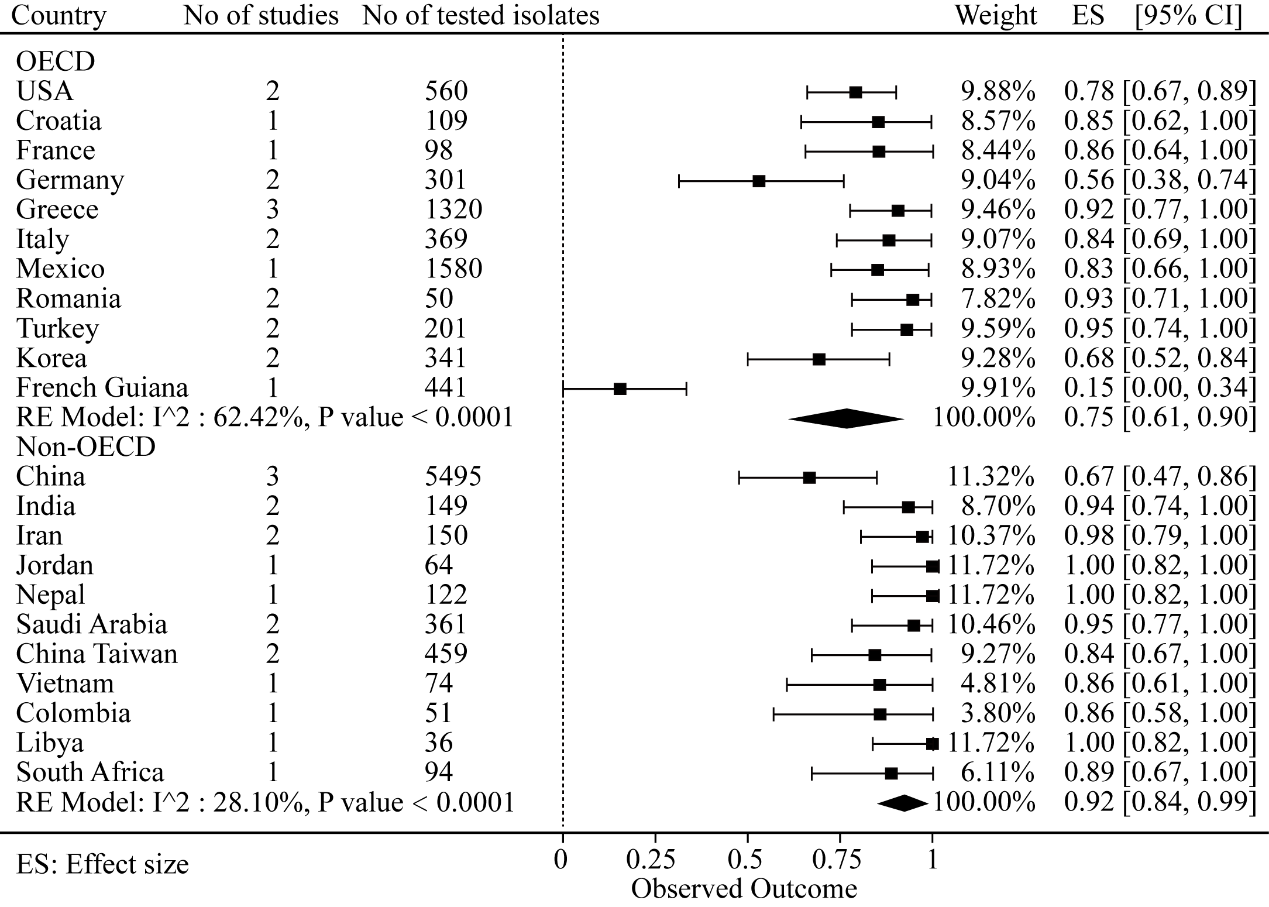


**Supplementary Figure S8:** The pooled prevalence of meropenem resistance in *A. baumannii* isolates from patients during 2006-2016, stratified by OECD status


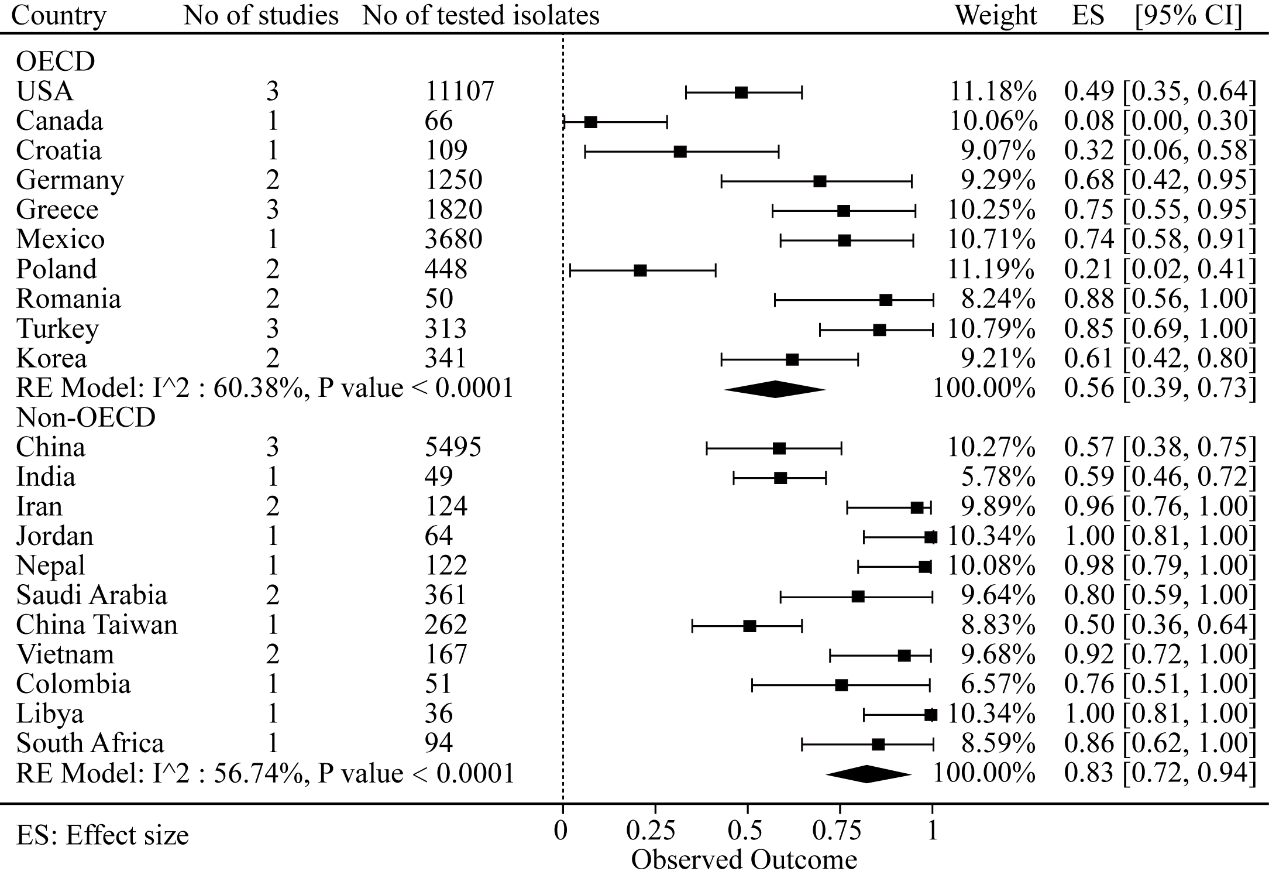


**Supplementary Figure S9:** The pooled prevalence of piperacillin-tazobactam resistance in *A. baumannii* isolates from patients, stratified by OECD status


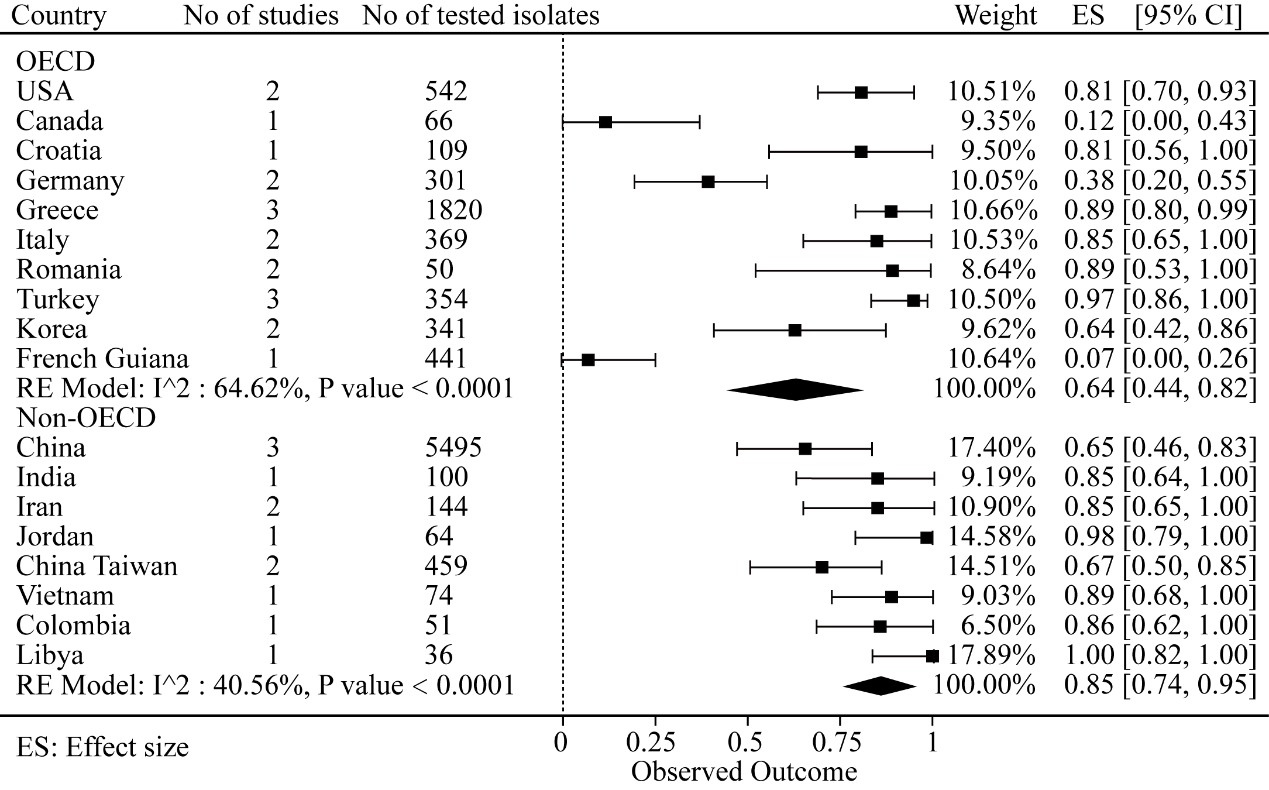


**Supplementary Figure S10:** The pooled prevalence of Cefepime resistance in *A. baumannii* isolates from patients during 2006-2016, stratified by OECD status


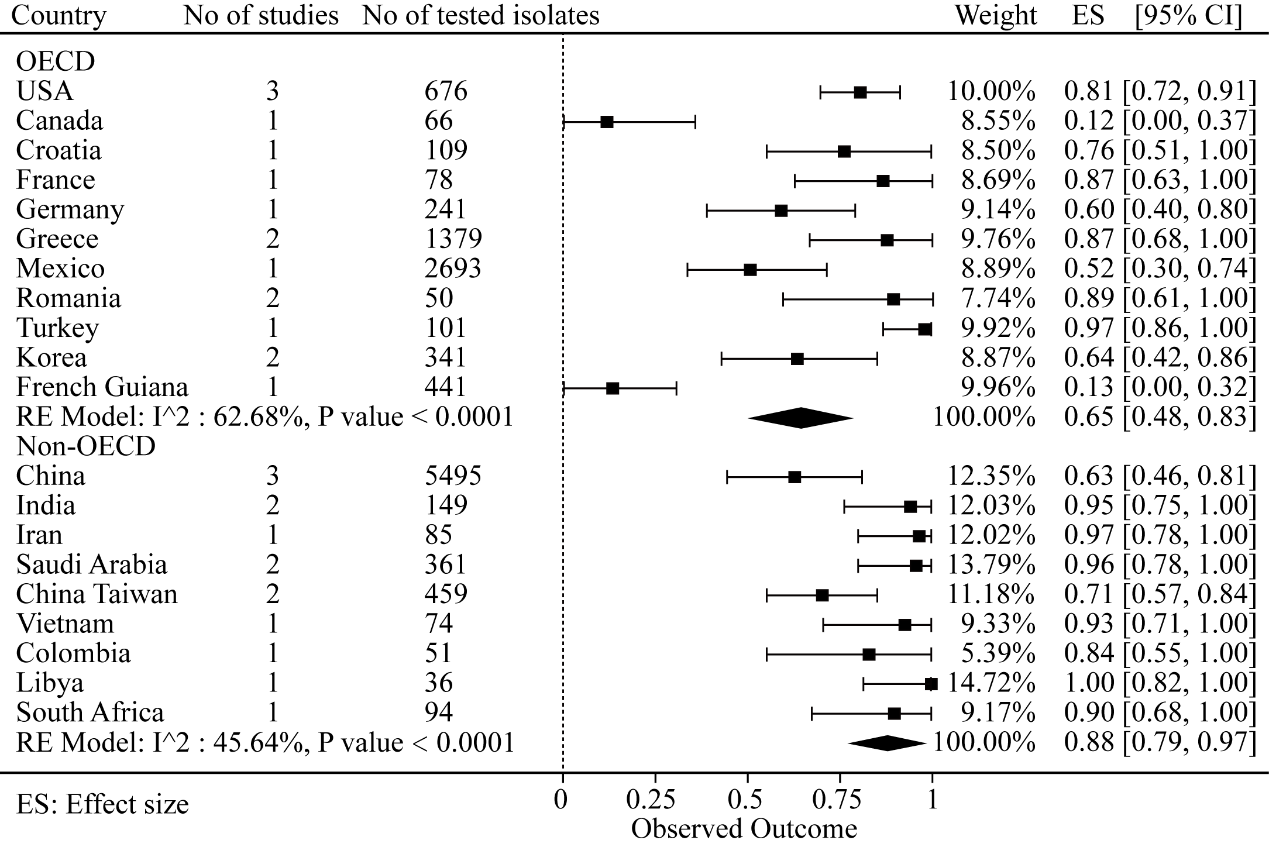


**Supplementary Figure S11:** The pooled prevalence of colistin resistance in *A. baumannii* isolates from patients during 2006-2016, stratified by OECD status.


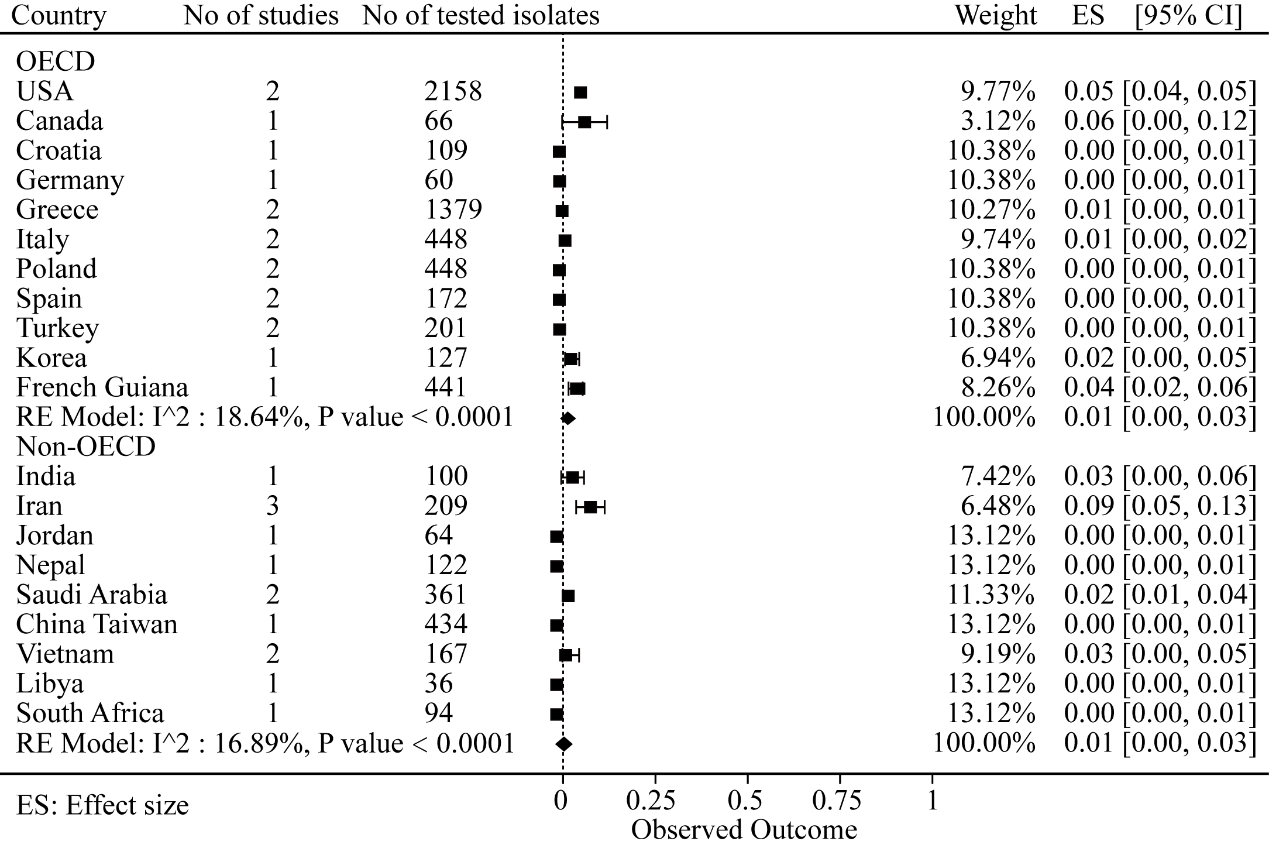


**Supplementary Figure S12:** The pooled prevalence of tigecycline resistance in *A. baumannii* isolates from patients during 200-2016, stratified by OECD status


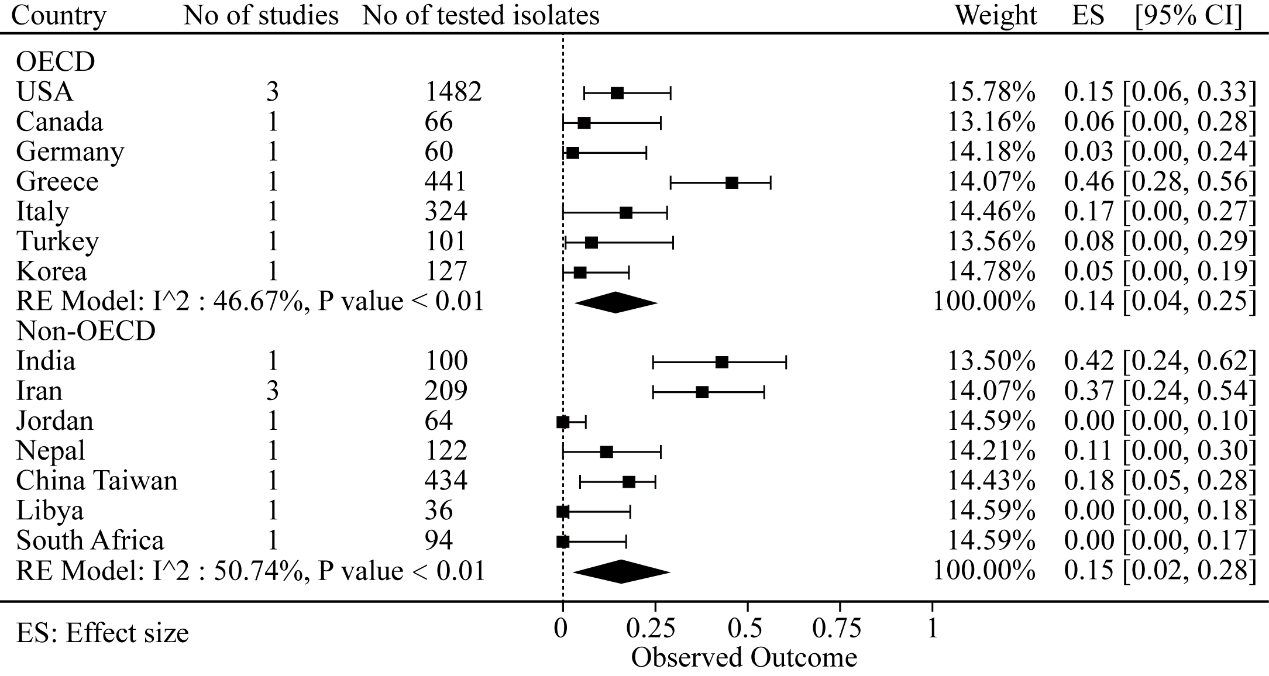


**Supplementary Figure S13:** The gap of resistance between OECD and non-OECD countries for each antibiotic during 2006-2010 and 2011-2016


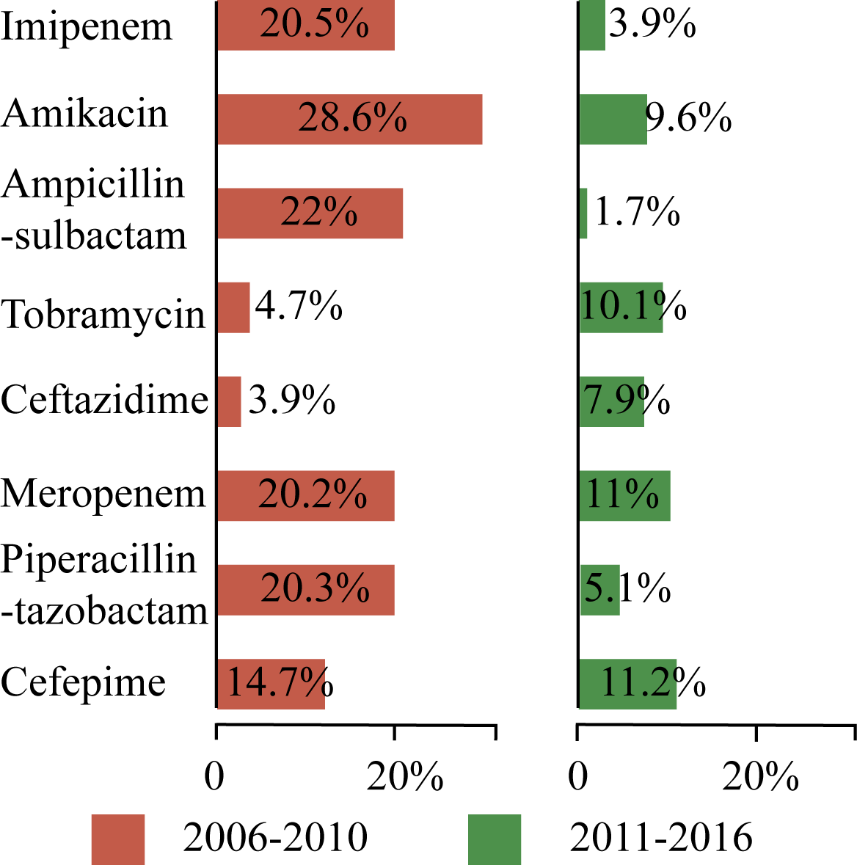


**Supplementary Figure S14:** The pooled mortality of MDR *A. baumannii* infections worldwide during 2000-2016^13, 25, 63-83^


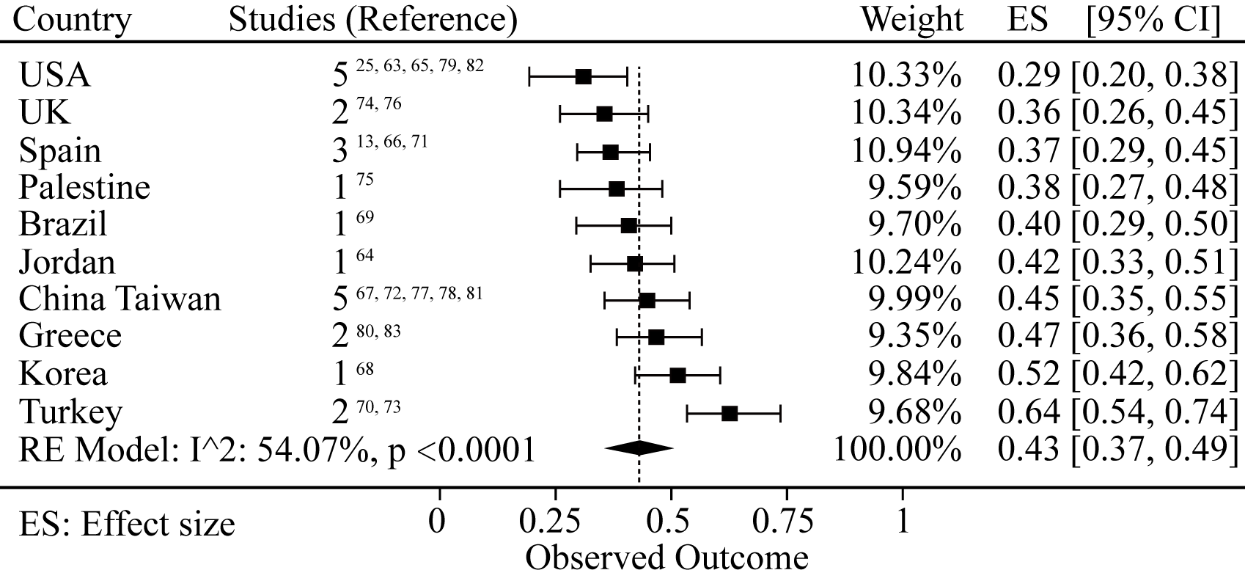


**Supplementary Reference:**

1 Koeleman JG, Stoof J, Van Der Bijl MW *et al*. Identification of epidemic strains of Acinetobacter baumannii by integrase gene PCR. *J Clin Microbiol* 2001; **39:** 8-13.

2 Sahm DF, Critchley IA, Kelly LJ *et al.* Evaluation of current activities of fluoroquinolones against gram-negative bacilli using centralized in vitro testing and electronic surveillance. *Antimicrob Agents Chemother* 2001; **45:** 267-274.

3 Martin-Lozano D, Cisneros JM, Becerril B *et al.* Comparison of a repetitive extragenic palindromic sequence-based PCR method and clinical and microbiological methods for determining strain sources in cases of nosocomial Acinetobacter baumannii bacteremia. *J Clin Microbiol* 2002; **40:** 4571-4575.

4 Karlowsky JA, Draghi DC, Jones ME *et al*. Surveillance for antimicrobial susceptibility among clinical isolates of Pseudomonas aeruginosa and Acinetobacter baumannii from hospitalized patients in the United States, 1998 to 2001. *Antimicrob Agents Chemother* 2003; **47:** 1681-1688.

5 Katragkou A, Kotsiou M, Antachopoulos C *et al.* Acquisition of imipenem-resistant Acinetobacter baumannii in a pediatric intensive care unit: A case-control study. *Intensive Care Med* 2006; **32:** 1384-1391.

6 Arda B, Sipahi OR, Yamazhan T *et al.* Short-term effect of antibiotic control policy on the usage patterns and cost of antimicrobials, mortality, nosocomial infection rates and antibacterial resistance. *J Infect* 2007; **55:** 41-48.

7 Ko KS, Suh JY, Kwon KT *et al.* High rates of resistance to colistin and polymyxin B in subgroups of Acinetobacter baumannii isolates from Korea. *J Antimicrob Chemother* 2007; **60:** 1163-1167.

8 Landman D, Bratu S, Kochar S *et al.* Evolution of antimicrobial resistance among Pseudomonas aeruginosa, Acinetobacter baumannii and Klebsiella pneumoniae in Brooklyn, NY. *J Antimicrob Chemother* 2007; **60:** 78-82.

9 Oteo J, Garcia-Estebanez C, Miguelanez S *et al.* Genotypic diversity of imipenem resistant isolates of Acinetobacter baumannii in Spain. *J Infect* 2007; **55:** 260-266.

10 Baran G, Erbay A, Bodur H *et al.* Risk factors for nosocomial imipenem-resistant Acinetobacter baumannii infections. *Int J Infect Dis* 2008; **12:** 16-21.

11 Dauner DG, May JR, Steele JC. Assessing antibiotic therapy for Acinetobacter baumannii infections in an academic medical center. *Eur J Clin Microbiol Infect Dis* 2008; **27:** 1021-1024.

12 Gbaguidi-Haore H, Legast S, Thouverez M *et al*. Ecological study of the effectiveness of isolation precautions in the management of hospitalized patients colonized or infected with Acinetobacter baumannii. *Infect Control Hosp Epidemiol* 2008; **29:** 1118-1123.

13 Rodriguez Guardado A, Blanco A, Asensi V *et al.* Multidrug-resistant Acinetobacter meningitis in neurosurgical patients with intraventricular catheters: assessment of different treatments. *J Antimicrob Chemother* 2008; **61:** 908-913.

14 van den Broek PJ, van der Reijden TJ, van Strijen E *et al*. Endemic and epidemic acinetobacter species in a university hospital: an 8-year survey. *J Clin Microbiol* 2009; **47:** 3593-3599.

15 Wadl M, Heckenbach K, Noll I *et al.* Increasing occurrence of multidrug-resistance in Acinetobacter baumannii isolates from four German University Hospitals, 2002-2006. *Infection* 2010; **38:** 47-51.

16 McCracken M, Mataseje LF, Loo V *et al.* Characterization of Acinetobacter baumannii and meropenem-resistant Pseudomonas aeruginosa in Canada: results of the CANWARD 2007-2009 study. *Diagn Microbiol Infect Dis* 2011; **69:** 335-341.

17 Maraki S, Mavros MN, Kofteridis DP *et al.* Epidemiology and antimicrobial sensitivities of 536 multi-drug-resistant gram-negative bacilli isolated from patients treated on surgical wards. *Surg Infect (Larchmt)* 2012; **13:** 326-331.

18 Park YK, Jung SI, Park KH *et al.* Changes in antimicrobial susceptibility and major clones of Acinetobacter calcoaceticus-baumannii complex isolates from a single hospital in Korea over 7 years. *J Med Microbiol* 2012; **61:** 71-79.

19 Samonis G, Maraki S, Vouloumanou EK *et al.* Antimicrobial susceptibility of non-fermenting Gram-negative isolates to isepamicin in a region with high antibiotic resistance. *Eur J Clin Microbiol Infect Dis* 2012; **31:** 3191-3198.

20 Dally S, Lemuth K, Kaase M et al. DNA microarray for genotyping antibiotic resistance determinants in Acinetobacter baumannii clinical isolates. *Antimicrob Agents Chemother* 2013; **57:** 4761-4768.

21 De Francesco MA, Ravizzola G, Peroni L *et al.* Prevalence of multidrug-resistant Acinetobacter baumannii and Pseudomonas aeruginosa in an Italian hospital. *J Infect Public Health* 2013; **6:** 179-185.

22 Metan G, Sariguzel F, Sumerkan B *et al*. Clonal diversity and high prevalence of OXA-58 among Acinetobacter baumannii isolates from blood cultures in a tertiary care centre in Turkey. *Infect Genet Evol* 2013; **14:** 92-97.

23 Morfin-Otero R, Alcantar-Curiel MD, Rocha MJ *et al.* Acinetobacter baumannii infections in a tertiary care hospital in Mexico over the past 13 years. *Chemotherapy* 2013; **59:** 57-65.

24 Cicek AC, Saral A, Iraz M *et al.* OXA- and GES-type beta-lactamases predominate in extensively drug-resistant Acinetobacter baumannii isolates from a Turkish University Hospital. *Clin Microbiol Infect* 2014; **20:** 410-415.

25 Fitzpatrick MA, Ozer E, Bolon MK *et al*. Influence of ACB complex genospecies on clinical outcomes in a U.S. hospital with high rates of multidrug resistance. *J Infect* 2015; **70:** 144-152.

26 Guzek A, Rybicki Z, Korzeniewski K, Mackiewicz K, Saks E, Chcialowski A*, et al.* Etiological factors causing lower respiratory tract infections isolated from hospitalized patients. *Adv Exp Med Biol* 2015; **835:** 37-44.

27 Kaliterna V, Kaliterna M, Hrenovic J, Barisic Z, Tonkic M, Goic-Barisic I. Acinetobacter baumannii in Southern Croatia: clonal lineages, biofilm formation, and resistance patterns. *Infect Dis (Lond)* 2015; **47**(12)**:** 902-907.

28 Spiliopoulou A, Jelastopulu E, Vamvakopoulou S *et al.* In vitro activity of tigecycline and colistin against A. baumannii clinical bloodstream isolates during an 8-year period. *J Chemother* 2015; **27:** 266-270.

29 Lazureanu V, Porosnicu M, Gandac C *et al.* Infection with Acinetobacter baumannii in an intensive care unit in the Western part of Romania. *BMC Infect Dis* 2016; **16 Suppl 1:** 95.

30 Mahamat A, Bertrand X, Moreau B *et al.* Clinical epidemiology and resistance mechanisms of carbapenem-resistant Acinetobacter baumannii, French Guiana, 2008-2014. *Int J Antimicrob Agents* 2016; **48:** 51-55.

31 Zilberberg MD, Kollef MH, Shorr AF. Secular trends in Acinetobacter baumannii resistance in respiratory and blood stream specimens in the United States, 2003 to 2012: A survey study. *J Hosp Med* 2016; **11:** 21-26.

32 Mezzatesta ML, D'Andrea MM, Migliavacca R *et al.* Epidemiological characterization and distribution of carbapenem-resistant Acinetobacter baumannii clinical isolates in Italy. *Clin Microbiol Infect* 2012; **18:** 160-166.

33 Principe L, Piazza A, Giani T *et al.* Epidemic diffusion of OXA-23-producing Acinetobacter baumannii isolates in Italy: results of the first cross-sectional countrywide survey. *J Clin Microbiol* 2014; **52:** 3004-3010.

34 Izdebski R, Fiett J, Hryniewicz W *et al*. Molecular analysis of Acinetobacter baumannii isolates from invasive infections in 2009 in Poland. *J Clin Microbiol* 2012; **50:** 3813-3815.

35 Bonnin RA, Poirel L, Licker M *et al*. Genetic diversity of carbapenem-hydrolysing beta-lactamases in Acinetobacter baumannii from Romanian hospitals. *Clin Microbiol Infect* 2011; **17:** 1524-1528.

36 Ben RJ, Yang MC, Hsueh JC *et al*. Molecular characterisation of multiple drug-resistant Acinetobacter baumannii isolates in southern Taiwan. *Int J Antimicrob Agents* 2011; **38:** 403-408.

37 Reguero MT, Medina OE, Hernandez MA *et al*. Antibiotic resistance patterns of Acinetobacter calcoaceticus-A. baumannii complex species from Colombian hospitals. *Enferm Infecc Microbiol Clin* 2013; **31:** 142-146.

38 Ruan Z, Chen Y, Jiang Y *et al.* Wide distribution of CC92 carbapenem-resistant and OXA-23-producing Acinetobacter baumannii in multiple provinces of China. *Int J Antimicrob Agents* 2013; **42:** 322-328.

39 Aly M, Tayeb HT, Al Johani SM *et al.* Genetic diversity of OXA-51-like genes among multidrug-resistant Acinetobacter baumannii in Riyadh, Saudi Arabia. *Eur J Clin Microbiol Infect Dis* 2014; **33:** 1223-1228.

40 Obeidat N, Jawdat F, Al-Bakri AG *et al*. Major biologic characteristics of Acinetobacter baumannii isolates from hospital environmental and patients' respiratory tract sources. *Am J Infect Control* 2014; **42:** 401-404.

41 Tan R, Liu J, Li M *et al*. Epidemiology and antimicrobial resistance among commonly encountered bacteria associated with infections and colonization in intensive care units in a university-affiliated hospital in Shanghai. *J Microbiol Immunol Infect* 2014; **47:** 87-94.

42 Bahador A, Raoofian R, Pourakbari B *et al*. Genotypic and Antimicrobial Susceptibility of Carbapenem-resistant Acinetobacter baumannii: Analysis of is Aba Elements and bla OXA-23-like Genes Including a New Variant. *Front Microbiol* 2015; **6:** 1249.

43 Elabd FM, Al-Ayed MS, Asaad AM *et al*. Molecular characterization of oxacillinases among carbapenem-resistant Acinetobacter baumannii nosocomial isolates in a Saudi hospital. *J Infect Public Health* 2015; **8:** 242-247.

44 Ku WW, Kung CH, Lee CH *et al.* Evolution of carbapenem resistance in Acinetobacter baumannii: an 18-year longitudinal study from a medical center in northern Taiwan. *J Microbiol Immunol Infect* 2015; **48:** 57-64.

45 Le Minh V, Thi Khanh Nhu N, Vinh Phat V *et al.* In vitro activity of colistin in antimicrobial combination against carbapenem-resistant Acinetobacter baumannii isolated from patients with ventilator-associated pneumonia in Vietnam. *J Med Microbiol* 2015; **64:** 1162-1169.

46 Lowings M, Ehlers MM, Dreyer AW *et al*. High prevalence of oxacillinases in clinical multidrug-resistant Acinetobacter baumannii isolates from the Tshwane region, South Africa - an update. *BMC Infect Dis* 2015; **15:** 521.

47 Modarresi F, Azizi O, Shakibaie MR *et al*. Iron limitation enhances acyl homoserine lactone (AHL) production and biofilm formation in clinical isolates of Acinetobacter baumannii. *Virulence* 2015; **6:** 152-161.

48 Rynga D, Shariff M, Deb M. Phenotypic and molecular characterization of clinical isolates of Acinetobacter baumannii isolated from Delhi, India. *Ann Clin Microbiol Antimicrob* 2015; **14:** 40.

49 Shrestha S, Tada T, Miyoshi-Akiyama T *et al.* Molecular epidemiology of multidrug-resistant Acinetobacter baumannii isolates in a university hospital in Nepal reveals the emergence of a novel epidemic clonal lineage. *Int J Antimicrob Agents* 2015; **46:** 526-531.

50 Tada T, Miyoshi-Akiyama T, Shimada K *et al.* Dissemination of clonal complex 2 Acinetobacter baumannii strains co-producing carbapenemases and 16S rRNA methylase ArmA in Vietnam. *BMC Infect Dis* 2015; **15:** 433.

51 Chatterjee S, Datta S, Roy S *et al.* Carbapenem Resistance in Acinetobacter baumannii and Other Acinetobacter spp. Causing Neonatal Sepsis: Focus on NDM-1 and Its Linkage to ISAba125. *Front Microbiol* 2016; **7:** 1126.

52 Mathlouthi N, El Salabi AA, Ben Jomaa-Jemili M *et al.* Early detection of metallo-beta-lactamase NDM-1- and OXA-23 carbapenemase-producing Acinetobacter baumannii in Libyan hospitals. *Int J Antimicrob Agents* 2016; **48:** 46-50.

53 Pourabbas B, Firouzi R, Pouladfar G. Characterization of carbapenem-resistant Acinetobacter calcoaceticus-baumannii complex isolates from nosocomial bloodstream infections in southern Iran. *J Med Microbiol* 2016; **65:** 235-239.

54 Qi L, Li H, Zhang C *et al.* Relationship between Antibiotic Resistance, Biofilm Formation, and Biofilm-Specific Resistance in Acinetobacter baumannii. *Front Microbiol* 2016; **7:** 483.

55 Koeleman JG, Stoof J, Biesmans DJ *et al*. Comparison of amplified ribosomal DNA restriction analysis, random amplified polymorphic DNA analysis, and amplified fragment length polymorphism fingerprinting for identification of Acinetobacter genomic species and typing of Acinetobacter baumannii. *J Clin Microbiol* 1998; **36:** 2522-2529.

56 Sahm DF, Marsilio MK, Piazza G. Antimicrobial resistance in key bloodstream bacterial isolates: electronic surveillance with the Surveillance Network Database--USA. *Clin Infect Dis* 1999; **29:** 259-263.

57 La Scola B, Gundi VA, Khamis A *et al*. Sequencing of the rpoB gene and flanking spacers for molecular identification of Acinetobacter species. *J Clin Microbiol* 2006; **44:** 827-832.

58 Quale JM, Landman D, Bradford PA *et al.* Molecular epidemiology of a citywide outbreak of extended-spectrum beta-lactamase-producing Klebsiella pneumoniae infection. *Clin Infect Dis* 2002; **35:** 834-841.

59 Landman D, Quale JM, Mayorga D *et al.* Citywide clonal outbreak of multiresistant Acinetobacter baumannii and Pseudomonas aeruginosa in Brooklyn, NY: the preantibiotic era has returned. *Arch Intern Med* 2002; **162:** 1515-1520.

60 Turton JF, Woodford N, Glover J *et al*. Identification of Acinetobacter baumannii by detection of the blaOXA-51-like carbapenemase gene intrinsic to this species. *J Clin Microbiol* 2006; **44:** 2974-2976.

61 Evans BA, Hamouda A, Towner KJ *et al*. OXA-51-like beta-lactamases and their association with particular epidemic lineages of Acinetobacter baumannii. *Clin Microbiol Infect* 2008; **14:** 268-275.

62 Jones RN, Ferraro MJ, Reller LB *et al*. Multicenter studies of tigecycline disk diffusion susceptibility results for Acinetobacter spp. *J Clin Microbiol* 2007; **45:** 227-230.

63 Zilberberg MD, Nathanson BH, Sulham K *et al*. Multidrug resistance, inappropriate empiric therapy, and hospital mortality in Acinetobacter baumannii pneumonia and sepsis. *Crit Care* 2016; **20:** 221.

64 Almomani BA, McCullough A, Gharaibeh R *et al*. Incidence and predictors of 14-day mortality in multidrug-resistant Acinetobacter baumannii in ventilator-associated pneumonia. *J Infect Dev Ctries* 2015; **9:** 1323-1330.

65 Goff DA, Bauer KA, Mangino JE. Bad bugs need old drugs: a stewardship program's evaluation of minocycline for multidrug-resistant Acinetobacter baumannii infections. *Clin Infect Dis* 2014; **59 Suppl 6:** S381-387.

66 Lopez-Cortes LE, Cisneros JM, Fernandez-Cuenca F *et al.* Monotherapy versus combination therapy for sepsis due to multidrug-resistant Acinetobacter baumannii: analysis of a multicentre prospective cohort. *J Antimicrob Chemother* 2014; **69:** 3119-3126.

67 Chuang YC, Cheng CY, Sheng WH *et al.* Effectiveness of tigecycline-based versus colistin- based therapy for treatment of pneumonia caused by multidrug-resistant Acinetobacter baumannii in a critical setting: a matched cohort analysis. *BMC Infect Dis* 2014; **14:** 102.

68 Shin JA, Chang YS, Kim HJ *et al.* Clinical outcomes of tigecycline in the treatment of multidrug-resistant Acinetobacter baumannii infection. *Yonsei Med J* 2012; **53:** 974-984.

69 Prata-Rocha ML, Gontijo-Filho PP, Melo GB. Factors influencing survival in patients with multidrug-resistant Acinetobacter baumannii infection. *Braz J Infect Dis* 2012; **16:** 237-241.

70 Tasbakan MS, Pullukcu H, Sipahi OR *et al*. Is tigecyclin a good choice in the treatment of multidrug-resistant Acinetobacter baumannii pneumonia? *J Chemother* 2011; **23:** 345-349.

71 Hernandez-Torres A, Garcia-Vazquez E, Gomez J *et al*. Multidrug and carbapenem-resistant Acinetobacter baumannii infections: Factors associated with mortality. *Med Clin (Barc)* 2012; **138:** 650-655.

72 Ye JJ, Lin HS, Kuo AJ *et al.* The clinical implication and prognostic predictors of tigecycline treatment for pneumonia involving multidrug-resistant Acinetobacter baumannii. *J Infect* 2011; **63:** 351-361.

73 Metan G, Sariguzel F, Sumerkan B. Factors influencing survival in patients with multi-drug-resistant Acinetobacter bacteraemia. *Eur J Intern Med* 2009; **20:** 540-544.

74 Gordon NC, Wareham DW. A review of clinical and microbiological outcomes following treatment of infections involving multidrug-resistant Acinetobacter baumannii with tigecycline. *J Antimicrob Chemother* 2009; **63:** 775-780.

75 Al Jarousha AM, El Jadba AH, Al Afifi AS *et al*. Nosocomial multidrug-resistant Acinetobacter baumannii in the neonatal intensive care unit in Gaza City, Palestine. *Int J Infect Dis* 2009; **13:** 623-628.

76 Enoch DA, Summers C, Brown NM *et al.* Investigation and management of an outbreak of multidrug-carbapenem-resistant Acinetobacter baumannii in Cambridge, UK. *J Hosp Infect* 2008; **70:** 109-118.

77 Lee NY, Lee HC, Ko NY *et al.* Clinical and economic impact of multidrug resistance in nosocomial Acinetobacter baumannii bacteremia. *Infect Control Hosp Epidemiol* 2007; **28:** 713-719.

78 Liao CH, Sheng WH, Chen YC *et al*. Predictive value of the serum bactericidal test for mortality in patients infected with multidrug-resistant Acinetobacter baumannii. *J Infect* 2007; **55:** 149-157.

79 Sunenshine RH, Wright MO, Maragakis LL *et al.* Multidrug-resistant Acinetobacter infection mortality rate and length of hospitalization. *Emerg Infect Dis* 2007; **13:** 97-103.

80 Betrosian AP, Frantzeskaki F, Xanthaki A *et al*. High-dose ampicillin-sulbactam as an alternative treatment of late-onset VAP from multidrug-resistant Acinetobacter baumannii. *Scand J Infect Dis* 2007; **39:** 38-43.

81 Kuo LC, Lai CC, Liao CH *et al.* Multidrug-resistant Acinetobacter baumannii bacteraemia: clinical features, antimicrobial therapy and outcome. *Clin Microbiol Infect* 2007; **13:** 196-198.

82 Roberts MC. Multidrug-resistant genes are associated with an 86-kb island in Acinetobacter baumannii. *Trends Microbiol* 2006; **14:** 375-378.

83 Falagas ME, Bliziotis IA, Siempos, II. Attributable mortality of Acinetobacter baumannii infections in critically ill patients: a systematic review of matched cohort and case-control studies. *Crit Care* 2006; **10:** R48.
